# Developing molecular tools and insights into the *Penstemon* genome using genomic reduction and next-generation sequencing

**DOI:** 10.1186/1471-2156-14-66

**Published:** 2013-08-08

**Authors:** Rhyan B Dockter, David B Elzinga, Brad Geary, P Jeff Maughan, Leigh A Johnson, Danika Tumbleson, JanaLynn Franke, Keri Dockter, Mikel R Stevens

**Affiliations:** 1 Plant and Wildlife Sciences Department, Brigham Young University, Provo, UT 84602, USA; 2 Biology Department, Brigham Young University, Provo, UT 84602, USA

**Keywords:** Breeding domesticated *Penstemon*, Genome reduction, Homologous sequences, LTR retroelements, Plantaginaceae, Pyrosequencing, Repetitive elements

## Abstract

**Background:**

*Penstemon’s* unique phenotypic diversity, hardiness, and drought-tolerance give it great potential for the xeric landscaping industry. Molecular markers will accelerate the breeding and domestication of drought tolerant *Penstemon* cultivars by, creating genetic maps, and clarifying of phylogenetic relationships. Our objectives were to identify and validate interspecific molecular markers from four diverse Penstemon species in order to gain specific insights into the *Penstemon* genome.

**Results:**

We used a 454 pyrosequencing and GR-RSC (genome reduction using restriction site conservation) to identify homologous loci across four *Penstemon* species (*P. cyananthus*, *P. davidsonii*, *P. dissectus,* and *P. fruticosus*) representing three diverse subgenera with considerable genome size variation. From these genomic data, we identified 133 unique interspecific markers containing SSRs and INDELs of which 51 produced viable PCR-based markers. These markers produced simple banding patterns in 90% of the species × marker interactions (~84% were polymorphic). Twelve of the markers were tested across 93, mostly xeric, *Penstemon* taxa (72 species), of which ~98% produced reproducible marker data. Additionally, we identified an average of one SNP per 2,890 bp per species and one per 97 bp between any two apparent homologous sequences from the four source species. We selected 192 homologous sequences, meeting stringent parameters, to create SNP markers. Of these, 75 demonstrated repeatable polymorphic marker functionality across the four sequence source species. Finally, sequence analysis indicated that repetitive elements were approximately 70% more prevalent in the *P. cyananthus* genome, the largest genome in the study, than in the smallest genome surveyed (*P. dissectus*).

**Conclusions:**

We demonstrated the utility of GR-RSC to identify homologous loci across related *Penstemon* taxa. Though PCR primer regions were conserved across a broadly sampled survey of *Penstemon* species (93 taxa), DNA sequence within these amplicons (12 SSR/INDEL markers) was highly diverse. With the continued decline in next-generation sequencing costs, it will soon be feasible to use genomic reduction techniques to simultaneously sequence thousands of homologous loci across dozens of *Penstemon* species. Such efforts will greatly facilitate our understanding of the phylogenetic structure within this important drought tolerant genus. In the interim, this study identified thousands of SNPs and over 50 SSRs/INDELs which should provide a foundation for future *Penstemon* phylogenetic studies and breeding efforts.

## Background

Interest is increasing in drought tolerant landscape plants due to water shortages experienced by many municipalities, especially in the Southwestern US [1,2]. However, the increased use of drought tolerant species also carries concerns regarding the introduction of non-native and potentially invasive species [3,4]. One way to address both issues is to landscape with native xeric flora [3]. *Penstemon* Mitchell (Plantaginaceae) has excellent potential for xeric landscapes and some *Penstemon* cultivars, adapted to mild climates, are already used throughout Europe as landscape plants [5-10]. Despite its potential, few *Penstemon* cultivars are used in xeric landscapes and there has been little to no drought or cold tolerant cultivar development for such landscapes [6-8,10-12]. *Penstemon*, with over 270 species, is one of the largest and most diverse plant genera of those that are strictly indigenous to North and Central America. This genus features a deep diversity in morphology, including a broad assortment of colors, flowers, and leaf structures. *Penstemon’s* putative center of origin is the arid Intermountain West of the United States [13,14] and has frequently been discussed as an untapped resource for xeric landscape cultivar development [5-7,9-11,15-17]. Because domestication and cultivar development, of any species, is slow, costly, and time consuming, few in the landscape industry have invested in native species breeding. However, given the recent and dramatic decrease in costs and relative ease of genotyping, we anticipate the wider utilization of marker assisted selection to accelerate breeding programs of native species, including drought tolerant *Penstemon*[18-20].

PCR-based markers are now essential tools to facilitate plant domestication, plant breeding, germplasm conservation, phylogenetics, and genetic mapping studies [19-22]. Not surprisingly, little molecular or traditional genetic work has been reported for *Penstemon*[23]. To achieve broad resolution of the genome with three of the most efficient markers, SSRs (simple sequence repeats or microsatellites), INDELs (insertions/deletions), and SNPs (single nucleotide polymorphisms), vast amounts of DNA sequence are needed, particularly for SNPs where sufficient read depth is needed to distinguish true polymorphisms from sequence noise [24-26]. With the development of next-generation sequencing (e.g., Roche 454-pyrosequencing) the cost of high-throughput marker discovery has been dramatically reduced [18]. Additionally, Maughan et al. [25] described a simple genome reduction method, known as GR-RSC (genome reduction using restriction site conservation), which reduces the genome by > 90% thereby, making it feasible to redundantly sequence the remaining genome with next-generation sequencing technologies. This process is repeated across multiple cultivars or species, with comparisons identifying many inter- and intraspecific homologous loci. Genomic reduction techniques consistently identify homologous loci between related species [20,27], and GR-RSC has enabled the identification and development of interspecific homologous SNPs [20].

We utilized GR-RSC to identify homologous sequences in four diploid (2*n* = 2*x* = 16) *Penstemon* species chosen to represent a range of taxonomic and genome size diversity [5,14]. Included in our analysis are two closely related species from the subgenus *Dasanthera* (*P. davidsonii* Greene and *P. fruticosus* (Pursh) Greene var. *fruticosus*), one from the subgenus *Habroanthus* (*P. cyananthus* Hook. var. *cyananthus*), and one (*P. dissectus* Elliot) from the monophyletic subgenus *Dissecti*, which is phenotypically divergent from all other *Penstemon* species. This experimental design allowed us to make broad inter- and intra-subgenera comparisons in *Penstemon*. The objectives of our study were three-fold: First, identify homologous SSR and INDEL markers from the four diverse species and test their conservation across 93, mostly xerophilic, *Penstemon* taxa. Second, identify conserved homologous sequences for SNPs for use in future interspecific studies. Third, assess observed variation in the GR-RSC sequences to gain insights into the *Penstemon* genome and possible reasons for the large size variation previously identified among the diploid taxa [5].

## Methods

### Plant material and DNA extraction

DNA from *P. cyananthus*, *P. davidsonii*, *P. dissectus*, and *P. fruticosus* leaf tissue was extracted using the CTAB purification method [28] with modifications [29] for the GR-RSC technique. The source localities and identification of these plants have been reported previously [5]. A single sample from each species with the highest quality and DNA concentration, as determined using a ND-1000 spectrophotometer (NanoDrop Technologies Inc., Montchanin, DE), was selected to provide the 500 ng of DNA necessary for the genome reduction protocol.

For the molecular marker experiments, we used 93 *Penstemon* taxa. Leaf tissue was collected mostly from wild populations in the United States Intermountain West (Table 1). Each field-collected sample was identified to species and (or) variety using taxonomic keys specific to the area [30,31]. We extracted DNA using Qiagen DNeasy Plant Mini Kit (Qiagen Inc., Valencia, CA), and concentrations were diluted to 25–35 ng/μL.

**Table 1:** ***Penstemon *taxa (with collection counties) utilized in the 12 marker analysis with respective marker sizes**

**Species**	**County**^**1**^	**Marker sizes in bp**											
		**PS004**	**PS011**	**PS012**	**PS014**	**PS017**	**PS032**	**PS034**	**PS035**	**PS048**	**PS052**	**PS053**	**PS075**
**Subgenus *Dasanthera***													
*P. davidsonii*	Purchased^2^	460	500	360	370	700	370	320, 950	520	440	220	320	140
*P. fruticosus* v. *fruticosus*	Purchased	460	500	360	410	700	340	360	520	420	220	320	140
*P. montanus* v*. montanus*	Utah	480	430	390	370	450	310	340, 310	470	430	200	390	115
**Subgenus *Dissecti***													
*P. dissectus*	Purchased	440	860	370	380	750	370	320	920	380	220	320, 450	140
**Subgenus *Habroanthus***													
*P. ammophilus*	Kane	480	800	400	430	470	300	1250, 340	470	420	230	360	125
*P. barbatus* v*. torreyi*	Garfield	420	800	400	490	500	320	340	500	410	200	360	110
*P. barbatus* v*. trichander*	San Juan	650, 480	850	420	500, 490	520	310	310	500	450, 410	200	370	130
*P. comarrhenus*	Garfield	650, 480	850	420	490	470	330, 310	310	500	430	200	360	125
*P. compactus*	Cache	440	850	400	500	490	300	300	480	410	210	390, 360	125
*P. cyananthus* v*. cyananthus*	Wasatch	420	860	400	410	750	370	310, 340	630	420	220	160, 320	160
*P. cyananthus* v*. subglaber*	Box Elder	440, 420	850	400	490, 470	500, 450	340, 310	310, 280	520	410	210	360	120
*P. cyanocaulis*	Emery	440	310	420	490, 470	520	330, 320	320	480	420	210	350	120
*P. eatonii* v*. eatonii*	Utah	420	800	420	490	450	320	300	500	NM^3^	210	350	135, 125
*P. eatonii* v*. undosus*	Washington	420	850	420	470	420	320	290	650, 500	410	210	340	125
*P. fremontii*	Uintah	480	850	400	430	490	320, 310	340	500	420	220	370	130
*P. gibbensii*	Daggett	480, 440	850	420	490	420	320	300	480	430	220	360	130
*P. idahoensis*	Box Elder	440	800	400	410	470	310	340	500	430	250	340	130
*P. laevis*	Kane	440	850	400	470	470	310	350, 320	500	420	220	360	125
*P. leiophyllus* v*. leiophyllus*	Iron	480	850, 490	420	430	450	310	340	480	430	220	350	120
*P. longiflorus*	Beaver	440	800	420, 400	470	470, 450	330, 310	310	500	450	230	350, 220	125
*P. navajoa*	San Juan	480	800	400	490	550	330, 300	360, 340	500	450	230	410	135, 130
*P. parvus*	Garfield	480	850	450	500, 490	490	320	300	500	430	210	380, 360	130
*P. pseudoputus*	Garfield	480	800	420, 400	430	490, 420	320	340	480	450	230, 220	350	130
*P. scariosus* v*. albifluvis*	Uintah	440	850	400	490	490	310	320	480	410	210	370	115
*P. scariosus* v*. cyanomontanus*	Uintah	440	850	400	490	490	330	310	500	420	210	360	115
*P. scariosus* v*. garrettii*	Duchesne	490, 480	850	420	430	490	320	420, 340	520	430	230	360	125
*P. scariosus* v*. scariosus*	Sevier	480	1500, 1300	400	470	520	340, 310	310	500	430	210	360	130
*P. speciosus*	Box Elder	440	800	420	500, 490	490	320	340, 310	520	310	210	360	120
*P. strictiformis*	San Juan	480	850	400	470	500, 470	370, 310	350	500	410	220	370	125
*P. strictus*	Wasatch	480	850	400	410	450	310	350	520, 500	430	230	340	110
*P. subglaber*	Sevier	480	850	420, 400	470	490	310	350	500	430	220	350	115
*P. tidestromii*	Juab	390	850	420	470	490	310	300	480	400	190	360	140, 120
*P. uintahensis*	Duchesne	480	850	420	490	450	340	300	520	410	220	380	120
*P. wardii*	Sevier	480	800	420	430	490, 450	310	310	520	420	220	340	135, 120
**Subgenus *Penstemon***													
*P. abietinus*	Sevier	440	AD^4^	390	400	520	320	340	500	430	230	350	125
*P. acaulis*	Daggett	570	490	420	430	470	320	350	480	420	220	340	120
*P. ambiguus* v. *laevissimus*	Washington	520	850	390	490	470	320	1250, 340	500	400	220	AD	120
*P. angustifolius* v. *dulcis*	Millard	440	850, 600	400	490	520	370, 150	310	520	420	220	360	125
*P. angustifolius* v. *venosus*	San Juan	480	310	390	470	470	320, 150	340	550	450	220	360	135
*P. angustifolius* v. *vernalensis*	Daggett	480	800	390	430	470	370, 150	350	500	420	220	380	125
*P. atwoodii*	Kane	440	800	420	490, 390	470	300	310	480	400	180	360, 280	115
*P. bracteatus*	Garfield	440	850	400	500	AD	330, 310	320	520	420	230	380	125
*P. breviculus*	San Juan	650, 480	190	400	AD	470	500, 220	320	480	430	210	350	125
*P. caespitosus* v. *caespitosus*	Uintah	440	850	390	390	490	320	NM	190	NM	210	390, 370	115
*P. caespitosus* v. *desertipicti*	Washington	440	230	390	470, 370	470, 360	330	350	1000, 300	430	210	400, 380	130
*P. caespitosus* v. *perbrevis*	Wasatch	420	490	390	430, 400	470	320	350	520	380	220	340	120
*P. carnosus*	Emery	440	850	420	490	490	330, 300	310	500	430	220	350	130, 120
*P. concinnus*	Beaver	440	800	420	430, 400	500	480	350	480	420	190	700, 360	120
*P. confusus*	Washington	480	850	420	490	520	300	320	480	450	220	350	125
*P. crandallii* v. *atratus*	San Juan	420	490	390	500	450	370	320	280	400	190	350	120
*P. crandallii* v. *crandallii*	San Juan	420	340, 190	390	500	450	370, 340	310	280	380	190	350	115
*P. deustus* v. *pedicellatus*	Teton	420	850	420	430	550	340	320	550	340	230	370	130
*P. dolius* v. *dolius*	Millard	NM	710	400	400	490, 320	530, 300	320	480	450, 420	180	340	105
*P. dolius* v. *duchesnensis*	Duchesne	420	AD	420	400	500	340	320	480	410	180	360, 340	140, 120
*P. eriantherus* v. *cleburnei*	Daggett	420	850	420	410	450	480	300	500	490, 420	190	390, 360	140, 130
*P. flowersii*	Uintah	480	AD	420, 400	490, 430	470	300	350	520	420	220	360	125
*P. franklinii*	Iron	480	800	400	430	470	320, 300	350	520	420	240	380	125
*P. goodrichii*	Uintah	420	650	390	400	490	480	310	480	400	200	370, 350	135
*P. grahamii*	Uintah	420	850	400	390	470	530, 320	350	500	420	230	500, 370	120
*P. humilis* v. *brevifolius*	Cache	390	850	370	500	450	340, 320	320	480	500	220	280	115
*P. humilis* v. *humilis*	Box Elder	420	850	390	410	520	330, 310	360	500	500, 470	220	350	120
*P. humilis* v. *obtusifolius*	Washington	420	800	390	520, 490	AD	330	340	480	470	200	350	120
*P. immanifestus*	Millard	480	710	420	490	380	300	320	480	410, 380	220	400, 360	120
*P. lentus* v. *albiflorus*	San Juan	NM	430	390	430	450	320	300	500	400	210	470, 370	140
*P. lentus* v. *lentus*	San Juan	480	850	400	430	470	300	310	500	410	210	400, 370	145
*P. linarioides* v. *sileri*	Washington	420	850	370	490, 390	470	330, 310	350	470	400	210	370	125
*P. marcusii*	Emery	390	800	450	370	490	310	340, 320	500	NM	200	390, 360	120
*P. moffatii*	Grand	390	800	420	390	490	330	340	480	430	290, 200	380, 350	140
*P. nanus*	Millard	480	800	420	390	470	280	320	470	NM	180	360	120
*P. ophianthus*	Sevier	520	850	420	370	900, 750	330, 310	310	480	420	190	AD	115
*P. pachyphyllus* v. *congestus*	Kane	480	850	400	430	470, 380	320	310	520	410	250	370	170
*P. pachyphyllus* v. *mucronatus*	Daggett	440	800	390, 370	430	500	320	300, 280	520, 500	430	220	350	120
*P. pachyphyllus* v. *pachyphyllus*	Duchesne	480	850	390	410	490	370, 330	340, 240	400, 190	500, 430	290, 230	380, 220	125
*P. palmeri* v. *palmeri*	Washington	440	850	400	500, 490	520, 490	330	310	500	430	210	380	125
*P. petiolatus*	Washington	420	1000	400	500, 490	500	330	300	480	420	210	380	145
*P. pinorum*	Washington	480	800	420	610	500	480	310	480	470	200	390	125
*P. procerus* v. *aberrans*	Garfield	440	1000, 850	450, 370	520	520	330	360	480	410	220	370	115
*P. procerus* v. *procerus*	Iron	420	850, 550	370	490	470	340, 310	360	470	470	220	340	120
*P. radicosus*	Daggett	420	AD	420	490	470	330, 310	310	500	450	200	360	125
*P. rydbergii* v. *aggregatus*	Box Elder	420	850	400	520	500	340	360	520	470	210	380	115
*P. rydbergii* v. *rydbergii*	Rich	420	710	400	520	500	370	320	500	470, 430	AD	390	115
*P. thompsoniae*	Kane	420	AD	370	500	450	340, 320	340	500	410	220	390, 370	130
*P. tusharensis*	Beaver	420	1300, 230	370	430	450	320	320	500, 300	410	230	340	120
*P. utahensis*	San Juan	480	410	420	430	490	300	310	500	410	220	370	125
*P. watsonii*	Sevier	420	AD	370	490	470	320	350	480	490	220	350	120
*P. whippleanus*	Iron	420	800	400	370	450	310	350	500	430	210	370	105
*P. yampaensis*	Daggett	570	710	400	430, 390	490	500, 320	310	480	410	260, 230	340	120
**Subgenus *Saccanthera***													
*P. leonardii* v*. higginsii*	Washington	390	1300	420, 400	490, 430	550, 520	320	310	480	470	250	AD	125
*P. leonardii* v*. leonardii*	Utah	440	800	420	430	490	320	340, 320	480	500	240	370	120
*P. leonardii* v*. patricus*	Tooele	440	850	370	470	AD	370	310	550, 520	470	230	380	115
*P. platyphyllus*	Salt Lake	420	800	400	430	470	330	310	520	430	240	AD	135
*P. rostriflorus*	Washington	420	1100, 430	400	410	420	320, 300	290	500	490, 430	470	370	120
*P. sepalulus*	Utah	420	800	400	470	450	330	310	500	430	230	390	130
**Total unique molecular weight bands**		**9**	**18**	**6**	**10**	**14**	**12**	**12**	**13**	**12**	**11**	**17**	**11**
**Total pairs of dual molecular weight bands**		**6**	**7**	**7**	**16**	**11**	**28**	**14**	**7**	**8**	**4**	**20**	**7**
**Total monomorphic markers**		**85**	**80**	**86**	**76**	**80**	**65**	**78**	**86**	**81**	**88**	**69**	**86**
**Total NM**		**2**	**0**	**0**	**0**	**0**	**0**	**1**	**0**	**4**	**0**	**0**	**0**
**Total AD**		**0**	**6**	**0**	**1**	**2**	**0**	**0**	**0**	**0**	**1**	**4**	**0**

^1^ All counties are in Utah except Teton, Co. which is in Wyoming.

^2^ Purchased = *P. davidsonii* and *P. fruticosus* were purchased from nurseries in Utah Co., Utah while *P. dissectus* was purchased from a nursery in Aiken Co., South Carolina.

^3^ NM = no marker.

^4^ AD = ambiguous data (usually multiple bands and or smearing).

### Genome reduction, barcode addition and 454 pyrosequencing

Genome reduction followed Maughan et al. [25]. Briefly, for each sample, *Eco*RI and *Bfa*I were used for the initial restriction digest, after which a biotin-labeled adapter was ligated to the *Eco*RI restriction site and a non-labeled adapter was ligated independently to the *Bfa*I restriction site. Next, a non-labeled size exclusion step using Chroma Spin + TE-400 columns (Clontech Laboratories, Inc., Mountain View, CA) and magnetic biotin-streptavidin separation (Dynabeads M-280 Streptavidin, Invitrogen Life Science Corporation, Carlsbad, CA) was performed. Unique multiplex identifiers (MID) barcodes were added independently to each species using primers complementary to the adapter and cut sites (Table 2). Preliminary amplification was performed using 95°C for 1 min., 22 cycles of 95°C for 15 s, 65°C for 30 s, and 68°C for 2 min. PCR products were loaded into a 1.2% agarose Flashgel DNA Cassette (Lonza Corporation; Rockland, ME) to verify smearing and adequate amplification in preparation for pyrosequencing.

**Table 2:** **The four multiplex identifiers (MID) barcodes (adapter) primers used for the genomes of *Penstemon cyananthus*, *P. dissectus*, *P. davidsonii*, and *P. fruticosus***

**Species**	**MID ID #**	***Eco*R1 MID primer**^**1**^	***Bfa*1 MID primer**^**2**^
*P. cyananthus*	MID 1	5′- ACGAGTGCGTGACTGCGTACCAATTC	5′- ACGAGTGCGTGATGAGTCCTGAGTA
*P. dissectus*	MID 2	5′- ACGCTCGACAGACTGCGTACCAATTC	5′- ACGCTCGACAGATGAGTCCTGAGTA
*P. davidsonii*	MID 3	5′- AGACGCACTCGACTGCGTACCAATTC	5′- AGACGCACTCGATGAGTCCTGAGTA
*P. fruticosus*	MID 4	5′- AGCACTGTAGGACTGCGTACCAATTC	5′- AGCACTGTAGGATGAGTCCTGAGTA

^1^ The “AATTC” at the 3′ end the primer was where adapters complement the enzyme *Eco*R1 cut site and the preceding “C” is where the base was changed to avoid further enzymatic cleavage of the fragment.

^2^ The “TA” at the 3′ end of the primer was where adapters complement the enzyme *Bfa*1 cut site and the preceding “G” is where the base was changed to avoid further enzymatic cleavage of the fragment.

After the initial PCR, concentrations of each of the four species samples were determined fluorometrically using PicoGreen® dye (Invitrogen, Carlsbad, CA). Samples were then pooled using approximately equal molar concentrations of each species except for *P. cyananthus* (genome size = 1C = 893 Mbp), where the molar concentration was doubled to maintain a similar genomic representation compared to the other three species with smaller genome sizes (*P. dissectus*, 1C = 462 Mbp; *P. davidsonii*, 1C = 483 Mbp; and *P. fruticosus*, 1C = 476 Mbp; [5]). DNA fragments between 500–600 bp were selected following Maughan et al. [25]. Sequencing was performed by the Brigham Young University DNA Sequencing Center (Provo, UT) using a half 454-pyrosequencing plate, Roche-454 GS GLX instrument, and Titanium reagents (Brandord, CT).

### Sequence assembly

Sequence data were sorted by species using their unique MID species barcode (Table 2) by means of the software package CLC Bio Workbench (v. 2.6.1; Katrinebjerg, Aarhus N, Denmark). Following sorting (Table 2), assemblies were performed using Roche’s de novo assembler, Newbler (v. 2.6), which yields consensus sequences (contigs) of all individual reads, from each independent species, for use in subsequent analyses.

A full assembly (all individual reads of all four species pooled together) was performed by Newbler with “complex genome” parameter set and a trim file with MID barcodes specified; all other parameters were left to their defaults. For all subsequent species assemblies (all individual reads of one species), these same parameters were used with a few added conservative options selected: an expected depth of ‘10’ (20 default), a minimum overlap length of ‘50’ (40 default), and a minimum overlap identity of 95% (90% default).

### Repeat element identification

Assembled sequences from all four species were masked for possible genome wide repetitive elements using a combination of RepeatModeler and RepeatMasker [32]. RepeatModeler is a de novo repeat element family identification and modeling algorithm that implements RECON [33] and RepeatScout [34]. RepeatModeler scanned all contigs from the four *Penstemon* species assemblies and produced a predicted repeat element library of predictive models to find repeat elements. Using this reference library, RepeatMasker then scanned the four species to filter out repetitive elements. Singletons were omitted from the analysis. To assess possible repetitive element biases with RepeatMasker when implementing a denovo library from RepeatModeler, we analyzed the GR-RSC data from *Arabidopsis* RIL’s (recombinant inbred lines) Ler-O and Col-4 from Maughan et al’s. [35] study, compared to the *Arabidopsis* non-reduced genome downloaded from TAIR (The Arabidopsis Information Resource) [36].

### Marker development, verification, and use

To identify SSRs, INDELs, and SNPs, we used software MISA and SNP_Finder_Plus (custom Perl-script), respectively [25,37,38]. RepeatMasker was used to identify and mask transposable elements. MISA parameters were set as follows: di-nucleotide motifs had a minimum of eight repeats, tri-nucleotide motifs had a minimum of six repeats, tetra-nucleotide motifs had a minimum of five repeats, and 100 bp was set as the interruption (max difference between two purported SSR alleles). For the comparison of SSR frequency and repeat motifs across species, “unmasked” assembly files were used to remove bias caused by masking low complexity reads. The following parameters were used to define the heuristic thresholds for SNP_Finder_Plus: 8× minimum read depth for the SNP, 30% proportion of the reads representing the minor allele and 90% identity (an indication of homozygosity within a single species used in a dual-species assembly) required for each SNP locus. These parameters also helped compensate for sequencing and assembly errors, which allow greater confidence in calling base pair discrepancies as actual SNPs in the dual-species assemblies and the confident identification of heterozygosity in the individual assemblies. For both individual assemblies and dual species assemblies SNPs reported are those conforming to the aforementioned parameters.

All genomic sequences matching the above criteria were used for marker development. Primer3 v2.0 [39] was used to identify primers for amplifying these markers, with the following parameters: optimal primer size = 20 (range = 18–27); product size range = 100–500 base bp; Tm range = 50–60°C with 55°C optimum; and maximum polynucleotide = 3. Allowing PCR products greater than 200 bp greatly increased the possibility of INDELs in the PCR products.

The PCR (SSR/INDEL) markers were validated using the original four species as template DNA. Each 10 μl PCR reaction had ~ 30 ng genomic DNA, 0.05 mM dNTPs, 0.1 mM cresol red, 1.0 μl of 10X PCR buffer (Sigma-Aldrich, St. Louis, MO), 0.5 units of JumpStart™ Taq DNA Polymerase (Sigma-Aldrich, St. Louis, MO) and 0.5 μM (each) of the forward and reverse primers. The thermal cycler (Mastercycler® Pro; Eppendorf International; Hamburg, Germany) was set as follows: 94°C for 30 s, 45 cycles of 92°C for 20 s, (primer specific annealing temperature)°C for 1 min. 30 s, 72°C for 2 min., and 72°C for 7 min. (final extension). Following PCR reactions, DNA was loaded into 3% Metaphor® agarose (Lonza Corporation; Rockland, ME) gels and run using a gel electrophoresis box at 100 V for 2 h. Optimal annealing temperatures for each SSR/INDEL marker were selected based on clarity of bands produced over varying annealing temperatures. Only SSR/INDEL markers with one or two reproducible bands are reported in the marker studies (Tables 1 and 3). The same conditions used for marker validation were used in the SSR/INDEL marker studies, except gel electrophoresis times were increased to 4 h at 100 V.

**Table 3:** Summary of marker characteristics including the primary SSR motif identified in the original GR-RSC (genome reduction using restriction site conservation) sequence, primer sequences, EFL (expected fragment length), total bands, and fragment sizes

**Marker name**^**1**^	**Primary motif**	**Forward primer (5′-3′)**	**GenBank accession ID**	**EFL**	**Total unique bands**	**Fragment size**			
		**Reverse primer (5′-3′)**				***P. cyananthus***	***P. davidsonii***	***P. dissectus***	***P. fruticosus***
PS003 ^(di,f)^	(AT)_8_	TGCCTCTGTCTTTACATTCCAA	JQ966997	217	3	360	260	250	260
		CATGAAGCACTGCAAATCCA							
PS004 ^(da,f)^	(ATT)_6_	TGTTTCAATTGCTGTCCACAT	JQ951613	476	3	420	460	440	460
		TTGTCTGTCCAAACGGTAGGT							
PS005 ^(c,di,f)^	(GAA)_6_	GCCCAACTTCCGTAATTGAA	JQ966998	303	3	260, 300	260	280	280
		AACTGCTTGCCACTCGACTC							
PS009 ^(c,da,f)^	(TGA)_6_	ACCTCGAACTTGACGGTCC	JQ966999	466	4	370, 650	540	650	600
		TTCTGAGGAGAAACCAAGGG							
PS011 ^(da,f)^	(GA)_8_	AAGTGCGACACTGGATGTCTT	JQ951614	435	2	860	500	860	500
		GCAGCTTCAGCTCCAGAAAT							
PS012 ^(c)^	(TA)_8_	TCCATATTGTAACCAACAATGACTG	JQ951615	402	3	400	360	370	360
		TGAATGGCAAACCGTAATCA							
PS013 ^(f)^	(TA)_8_	GAAGAATTGATTTAAACAAGATGCAA	JQ967000	399	2	400	650	650	400
		TCAGTACGTGAGAAACTTGATCAATAA							
PS014 ^(c)^	(TGA)_6_	CGATTTGGTATAGTTGGATTACGA	JQ951616	409	3	410	370	380	410
		CCTTCATCACCCGGTACTTG							
PS015 ^(di)^	(TCG)_6_	GCCGAGTTTCAAGAAAGCAA	JQ967001	409	2	490	500	490	490
		AATTACGACCTGCCACGC							
PS016 ^(c,di)^	(CT)_8_	CATGGCCCTTTCTTCACACT	JQ967002	447	3	NM^2^	1,100	1,060	1,030
		GACGCGGTTGGCTATACAGT							
PS017 ^(da,di)^	(AG)_9_	GAAGGCTTAGCATAAATCCTCAAA	JQ951617	455	2	750	700	750	700
		ATTAGGCTCCCACGAACAAA							
PS019 ^(c,di)^	(AG)_8_	AATCCCACAGCCCATACAAA	JQ967003	473	1	380	380	380	380
		TGAATTGAGTCCTATACCCTATTTCAA							
PS021 ^(f)^	(CT)_8_	CTTTAGCTTAGCTGGAATACACGTT	JQ967004	386	3	350	450	450	420
		AGATTCTTGCATCACAGTTCAATTA							
PS023 ^(da)^	(AG)_8_	GCTGGAGAATAACATGGCG	JQ967005	469	4	310	480	120, 740	480
		CCATCTTGCAAGTCCATACG							
PS024 ^(da,f)^	(CTG)_6_	CTTCTTGCCCTGTGCCTCT	JQ967006	403	2	430	430	400	430
		CCACCACCAACAACAACAAC							
PS025 ^(c,di)^	(TC)_9_	GCACATGAATGAAGGAATGC	JQ967007	440	3	440	410	440	400
		ACGATCTGTGAAGGAACCCA							
PS026 ^(c,da,di,f)^	(CTT)_6_	ACTTAATAATGCCTCCTTGTGTCA	JQ967008	465	1	460	NM	460	460
		TTCCGCAACGTTGTATTTGA							
PS028 ^(di)^	(AC)_9_	GGGAGGCAGGTAACAACAAA	JQ967009	316	4	950	400, 460	320	400, 460
		TACCTCTGCCGAACTGGATT							
PS029 ^(di)^	(TA)_8_	ACCAAGTTGTTGGATGTTTGG	JQ967010	440	3	840	500	500	420
		GGTTTGGAATGAGACTTAGAAGGA							
PS032 ^(c,di)^	(GT)_9_	ACAAAGTCTCCTCAATCGCC	JQ951618	328	2	370	370	370	340
		GCATGTACCGTGCACACACT							
PS034 ^(c)^	(AC)_9_	CCAAACAAATCAAACAGCACTC	JQ951619	322	5	310, 340	320, 950	320	360
		CATGCGAATCAGTGTTGCTAA							
PS035 ^(da,f)^	(TC)_9_	TTGCACAGCTACTTTGGCAT	JQ951620	486	3	630	520	920	520
		ATCTGTCCAAGGCATGGAAT							
PS036 ^(c,di)^	(TA)_8_	TTCCTAATTTGGTAGCTGCAATC	JQ967011	405	3	770	770, 820	590	770
		TCCGAGGAACTATTGCCATT							
PS038 ^(c,da)^	(TA)_8_	GTAATTACTTCGGCAGTTTGTTAATTT	JQ967012	100	1	NM	100	100	NM
		GGTGCGACCTAATTACGTTTCTAT							
PS040 ^(da)^	(CA)_9_	TAAAGAGGCTTAAGCGCGG	JQ967013	399	3	380	390	410	390
		ACCTGAAGAGCTGCGGAGTA							
PS041 ^(c,da,di,f)^	(AT)_8_	TTTCCGCAAGAGAAGAGCAT	JQ967014	249	3	270	670	270	240
		CTTGTGCACGATTCCATTGT							
PS045 ^(c,da)^	(CT)_8_	GCCACATACATGAAACGTGAA	JQ967015	366	4	460	NM	440	120, 400
		CGAACTCTCTTGTGTTTCTCCC							
PS047 ^(c,di,f)^	(AC)_8_	ACACGACATCGTTTCAGCAA	JQ967016	428	3	470, 510	440	470	470
		GCGTATGGAGAGATTTGGGA							
PS048 ^(c,di)^	(CA)_9_	GCATTAGATGCCGAAATATCTACAA	JQ951621	436	3	420	440	380	420
		TGCCTGTAGGTTGATTTCCTTT							
PS049 ^(c,da,di,f)^	(AG)_8_	CCCATCAATAAAGAAAGAAAGAAAGA	JQ967017	436	2	460	460	1,000	460
		GGTGAAACCCTGTCCTAAACC							
PS050 ^(c,di)^	(AT)_9_	GTGTAACCTCTGAACAAGTTTACTGAA	JQ967018	434	2	480	460	480	460
		TGCAGTGAGCCATGCTATTC							
PS051 ^(c,di,f)^	(TG)_8_	TGTAACACGACAATTTAACTCTTTCA	JQ967019	352	1	280	NM	280	280
		CGAGAACTCTTTCCGAGAACC							
PS052 ^(c,da,di,f)^	(AC)_9_	CGCGGTCAATCTTGAAATCT	JQ951622	206	1	220	220	220	220
		TGACTTCCTCTCTCTCTCTCACAC							
PS053 ^(di)^	(AC)_8_	AATCATAGTCTCGAGCGCGT	JQ951623	410	3	160, 320	320	320, 450	320
		GAGATAAATTAGATCAGCGCATCA							
PS054 ^(c,da,f)^	(GA)_8_	TCGTTAAGCAATCTCGGAGC	JQ967020	192	3	200	200	180	190
		TCGACTGGAGAGCAAAGCA							
PS055 ^(f)^	(AG)_8_	TGTGGTCCGGTTCCATAAAC	JQ967021	412	4	960	500	1,040	470
		TTTGTCTCCCTAATATGTGTGATGAT							
PS056 ^(da,di)^	(TG)_8_	CATGTTTCAGGATTGGGCTT	JQ967022	319	4	690	450	230	340
		CGGTTACACACAGGTTGTTGA							
PS057 ^(da,f)^	(AT)_8_	TGCCTAATGGACCTGATCCT	JQ967023	402	2	570	440	570	440
		CCCAATTGTTTGAAGAAAGAACA							
PS058 ^(da)^	(AT)_9_	GTGCAACCAATGCAACTAATTC	JQ967024	469	1	NM	720	NM	720
		TCTCTCATTTCCAATGATTTCTCA							
PS059 ^(di)^	(CT)_8_	CATCAATTGACACACAAGCAGA	JQ967025	312	2	930	340	340	340
		TCGAATCTTAAAGAAACACATCCA							
PS060 ^(c,di)^	(AC)_9_	CCATGAGAAGTAGATGACTGGGA	JQ967026	484	2	560	560	560	540
		TTGTAATTATGATTAACTTCCCTCGTT							
PS061 ^(da,f)^	(TA)_8_	CGACCAATCATCAACCAACA	JQ967027	453	3	480	480, 530	450, 480	NM
		GACGGGCAGAATAATTGGAA							
PS062 ^(c,di)^	(TA)_9_	TGGAGAGGGTACGAAAGTGC	JQ967028	320	2	350	290	350	290
		CAACGATCGATTATTAGCACCA							
PS064 ^(c,da,f)^	(AG)_8_	ATGGATGCCCTATGGGTACA	JQ967029	437	4	490	500, 680	470	470
		TGAAATGGAGGGAGTAATATAAACAA							
PS066 ^(di)^	(GA)_9_	CAAGGATGCAGGCTCTCATT	JQ967030	434	2	250	480	250, 480	480
		CTCTGCTCGTCGTAGTGCAA							
PS068 ^(c,da,di,f)^	(GA)_8_	TTTGGGATGCATTTCTCCAC	JQ967031	463	2	500	500	480	480
		TCAAAGTGACATCTTCCAACAAA							
PS069 ^(di)^	(GT)_8_	CATTGGGTCAGATTTGGCTT	JQ967032	309	4	220	210	390	350
		GCTTTCAGTTTGTATATTTGTGCC							
PS071 ^(c,di)^	(AT)_8_	AAGATGGCCCTGATCTGTTG	JQ967033	446	1	NM	NM	490	NM
		TTCGTGGGAGTTGCAAATTA							
PS074 ^(c,da,di,f)^	(AAG)_6_	AGAAATCTCGCTCTCCACGA	JQ967034	168	1	170	170	170	170
		CGACAACCTTAGTGATCGCTTT							
PS075 ^(c,da,di,f)^	(TA)_8_	CACCACTTTCGCAGCATTTA	JQ951624	120	2	160	140	140	140
		CAAATTACATTATTGTATGGAAACACG							
PS076 ^(c,di)^	(GTG)_6_	CTGACAGCAACATGAACATGAA	JQ967035	161	1	170	170	170	170
		CAATCTTTGCCAATTTCCCA							

^1^ Parentheses indicates the species possessing sequence from which primers were designed (c = *P. cyananthus*, da = *P. davidsonii*, di = *P. dissectus*, f = *P. fruticosus*).

^2^ NM = no marker.

The gels were evaluated and scored as: 1 = marker present; 0 = marker absent based upon molecular weight. The results were then analyzed to assess the strength of hierarchical signal in these data using 10,000 replications of fast bootstrapping as implemented in PAUP* v. 4.0b10 [40].

Our interspecific SNP genotyping was accomplished using Fluidigm (Fluidigm Corp., South San Francisco, CA) nanofluidic Dynamic Array Integrated Fluidic Circuit (IFC) Chips [40] on the EP-1TM System (Fluidigm Corp., South San Francisco, CA) and competitive allele-specific PCR KASPar chemistry (KBioscience Ltd., Hoddesdon, UK). A 5 μL sample mix, consisting of 2.25 μL genomic DNA (20 ng μL^-1^), 2.5 μL of 2x KBiosciences Allele Specific PCR (KASP) reagent Mix (KBioscience Ltd.), and 0.25 μL of 20x GT sample loading reagent (Fluidigm Corp., South San Francisco, CA) was prepared for each DNA sample. Similarly, a 4 μL 10x KASP Assay, containing 0.56 μL of the KASP assay primer mix (allele specific primers at 12 μM and the common reverse primer at 30 μM), 2 μL of 2x Assay Loading Reagent (Fluidigm Corp., South San Francisco, CA), and 1.44 μL DNase-free water was prepared for each SNP assay.

The two assay mixes were added to the dynamic array chip, mixed, and then thermal cycled using an integrated fluidic circuit Controller HX and FC1 thermal cycler (Fluidigm Corp., South San Francisco, CA). The thermo cycler was set as follows: 70°C for 30 min; 25°C for 10 min for thermo mixing of components followed by hot-start Taq polymerase activation at 94°C 15 min then a touchdown amplification protocol consisting of 10 cycles for 94°C for 20 sec, 65°C for 1 min (decreasing 0.8°C per cycle), 26 cycles of 94°C for 20 sec, 57°C for 1 min, and then hold at 20°C for 30 sec. Five end-point fluorescent images of the chip were acquired using the EP-1TM imager (Fluidigm Corp., South San Francisco, CA), once after the initial touchdown cycles were complete and then after each additional run on “additional touchdown cycles.” The extra cycles were run four times, with an analysis of the chip after each run.

The determination of each SNP allele was based on a minimum of at least two of three SNP genotyping experiments. The primers were then analyzed for functionality using the results from each of the five stops for each chip, which were compared to determine the most accurate call. Functionality was determined by number of calls verses no calls, and consistency.

### Cross species sequencing verification

To evaluate the DNA sequence homology and polymorphism type (SSR or INDEL) at specific marker amplicons (Table 1) across the *Penstemon* genus, DNA samples from each of five species (*P. cyananthus*, *P. davidsonii*, *P. dissectus*, *P. fruticosus*, and *P. pachyphyllus*) were amplified and Sanger sequenced. We accomplished the PCR amplification using Qiagen HotStarTaq Plus Master Mix (Valencia, CA, USA) according to the manufacturer’s recommendations. The amplification protocol consisted of an initial denaturation step of 5 min at 95°C, followed by 40 cycles of amplification consisting of 30 sec denaturation at 94°C, 30 sec for primer annealing at 55°C and 1 min of extension at 72°C. PCR products were separated on 1% agarose gels run in 0.5X TBE and visualized by ethidium bromide staining and UV transillumination. PCR products were purified using a standard ExoSAP (Exonuclease I/Shrimp Alkaline Phosphatase) protocol and sequenced directly as PCR products. DNA sequencing was performed at the Brigham Young University DNA Sequencing Center (Provo, UT, USA) using standard ABI Prism Taq dye-terminator cycle- sequencing methodology. DNA sequences were analyzed, assembled and aligned using Geneious software (Biomatters, Auckland, New Zealand).

### Gene ontology

We used BLASTX [41] on assembled sequences of all four species to compare with the GenBank refseq-protein database [42] with a threshold of < 1.0e^-15^. Blast2GO (v2.4.2) was used to map the blast hits and annotate them to putative cellular components, biological processes, and molecular functions found in the blast database [43]. For species comparisons, the GO level 3 was used for cellular components and level 2 was used for both biological processes and molecular functions.

Assembled sequences of all four species were also compared to all available *Antirrhinum* and *Mimulus* (genera more or less related to *Penstemon*) genes on GenBank (downloaded 23 June 2011). Comparisons were made using BLASTN [41] with an e-value threshold of <1.0e^-13^.

## Results and discussion

### Genome reduction, pyrosequencing and species assemblies

Given that a full 454 pyrosequencing plate using Titanium reagents is capable of producing 1.3 million reads averaging ~400 bp each [25], we expected a half plate to produce approximately 250 Mbp from 650,000 reads. Our reaction produced 287 Mbp from 733,413 reads, 20% more than expected, with an average read length of 392 bp. In total, 93.8, 46.4, 48.8, and 53.3 Mbp were sequenced from *P. cyananthus*, *P. dissectus*, *P. davidsonii* and *P. fruticosus*, respectively, closely resembling the 2:1:1:1 ratio of DNA pooled from each species for sequencing (Table 4). Likewise, from our de novo assemblies, we identified nearly twice as many contigs, 9,714 in *P. cyananthus* than the 4,777 found in *P. fruticosus*, for example, which was expected because we sequenced approximately twice as much DNA from *P. cyananthus* than the other three species. There was 0.6% of *P. cyananthus* genome represented compared to 0.5% average coverage of the other three species (Table 4); thus, essentially an equal genome representation from each species was realized using the GR-RSC technique by pooling approximately equal genome molar concentrations in the sequencing reaction. The contigs of this study have been deposited at DDBJ/EMBL/GenBank as a Whole Genome Shotgun project under the accessions AKKG00000000 (*P. cyananthus*), AKKH00000000 (*P. dissectus*), AKKI00000000 (*P. davidsonii*), and AKKJ00000000 (*P. fruticosus*). The version described in this paper is the first version for each accession, XXXX01000000.

**Table 4:** **Summary data from 454-pyrosequencing and Newbler de-novo assembly (v.2.0.01) of *Penstemon cyananthus*, *P. dissectus*, *P. davidsonii*, and *P. fruticosus***

**Assembly**	**Genome size (Mbp)**^**1**^	**GC content**	**Reads**	**Bases**^**2**^	**% Reads assembled**	**% Bases assembled**	**Contigs created**	**Bases in assembly**	**% Genome represented**	**Average coverage**	**Bases shared between assemblies**
*P. cyananthus*	893	36.4%	199,329	87,753,792	53.1%	50.0%	9,714	4,623,755	0.5%	7.7X	
*P. dissectus*	462	34.5%	98,868	43,304,550	52.8%	50.9%	5,364	2,629,819	0.6%	8.2X	
*P. davidsonii*	483	35.3%	103,963	45,599,742	45.8%	43.5%	4,882	2,376,141	0.5%	9.1X	
*P. fruticosus*	476	35.2%	113,146	49,786,980	41.0%	38.8%	4,777	2,322,606	0.5%	8.9X	
*P. cyananthus* × *P. dissectus*			298,197	131,058,342	53.0%	50.1%	14,523	6,915,079			338,495
*P. cyananthus* × *P. davidsonii*			303,292	133,353,534	49.9%	46.9%	14,254	6,757,023			242,873
*P. cyananthus* × *P. fruticosus*			312,475	137,540,772	47.8%	44.9%	14,134	6,705,536			240,825
*P. dissectus* × *P. davidsonii*			202,831	88,904,292	48.3%	46.1%	10,053	4,855,491			150,469
*P. dissectus* × *P. fruticosus*			212,014	93,091,530	45.7%	43.5%	9,873	4,774,539			177,886
*P. davidsonii* × *P. fruticosus*			217,109	95,386,722	44.0%	41.7%	9,184	4,442,194			256,553
Full Penstemon Assembly			730,215	265,987,500	47.9%	46.4%	44,966	16,363,589			

^1^ The diploid (2n = 2x = 16) genome size as reported by Broderick et al. [5].

^2^ Bases denotes the total number of bases used to create the assembly and not the total number of bases sequenced.

DNA sequences produced by the GR-RSC technique represent a broad sample of the genome. With this sample, we can begin to estimate genome-wide characteristics, such as GC content, frequency of repeat elements, and so forth. From the genome reduction, GC content was measured to be 36.4%, 34.5%, 35.3%, and 35.15% for *P. cyananthus*, *P. dissectus*, *P. davidsonii* and *P. fruticosus,* respectively (Table 4), matching the average 35% GC content reported for dicots [44]. Using the dicot average GC content a priori, we estimated a theoretical frequency of the *Bfa*I and *Eco*RI recognition sites. The theoretical GC content in combination with estimated genome sizes of the four species [5] suggested the GR-RSC should have rendered a 104 fold reduction of the genome of each species. With a reduced genome of these species, the 650,000 reads that were sequence suggest an average of 11× coverage; however the observed read depth was 8.5×, 22.7% less than expected (Table 4). This lighter coverage is partly due to the lower than expected specificity of reads. An average of 48.2% of the reads were matched to contigs with the other 51.8% either too short or lacking in homology to successfully match to a contig (Table 4).

The full assembly of all four *Penstemon*, using the Newbler de novo assembler, produced a total of 44,966 contigs, representing 16.4 Mbp, or 5.7% of our total sequence. In the individual species assemblies of *P. cyananthus*, *P. dissectus*, *P. davidsonii*, and *P. fruticosus*, a total of 9,714, 5,364, 4,882, and 4,777 contigs were created representing 4.6, 2.6, 2.4, and 2.3 Mbp of assembled bases respectively. These contigs represent, on average, 0.5% of the total genomes being sequenced (Table 4).

### Marker analysis

We utilized assembly contigs from genomic sequence of all four species with “masked” multiple repeats, such as transposons, to identify SSRs. *Penstemon cyananthus*, *P. dissectus*, *P. davidsonii*, and *P. fruticosus* had 97, 113, 49, and 58 SSRs identified respectively (Table 5). There were more SSRs identified in *P. dissectus* than *P. cyananthus,* which has a 1.9 times larger genome and a higher representation of sequence than *P. dissectus* (Table 5). This inverse relationship between genome size and SSRs content agrees with observations in other plant genomes [45]. Some SSRs were found as putative homologs in multiple species; after eliminating redundancies, we tallied 133 unique SSRs (Table 3). We generated primer pairs surrounding 77 of these SSRs large enough to potentially capture INDELs, of these, 51 produced 1 or 2 reproducible bands with no or few faint superfluous bands. From those 51, there was an overall success rate of 94% with 42 (82%) being polymorphic between the four species (Table 3).

**Table 5:** Data obtained from MISA (SSR), Blast2GO (GO) and RepeatMasker (RM)

			***Penstemon *species**			
			***P. cyananthus***	***P. dissectus***	***P. davidsonii***	***P. fruticosus***
SSR	Total SSRs^1^		97	113	49	58
	SSRs/Assembly Length		2.1E-05 (~1/48000)	4.3E-05 (~1/23000)	2.1E-05 (~1/48000)	2.5E-05 (~1/40000)
	Repeat Type	di-	44.3%	40.7%	46.9%	48.3%
		tri-	45.4%	43.4%	44.9%	41.4%
		tetra-	10.3%	15.9%	8.2%	10.3%
GO	Contigs Analyzed		9,714	5,364	4,882	4,777
	Blast Hits Found^2^		1,899	1,125	1,121	1,091
	Annotated Hits		1,430	844	388	826
	% Blast Hits		19.5%	21.0%	23.0%	22.8%
	% Annotated		14.7%	15.7%	7.9%	17.3%
RM	Masked Repeat Elements		28.5%	16.8%	17.4%	16.1%
	Retroelements (LTR)		7.8%	3.0%	4.9%	4.6%
	DNA Transposons		0.3%	0.9%	1.0%	1.0%
	Other Repeats^3^		20.4%	12.9%	11.6%	10.5%

^1^ For MISA, “unmasked” individual species assemblies were used.

^2^ Sequence compared to the GenBank refseq-protein database e-value threshold of <1.0e^-15^.

^3^ Other Repeats includes: lines, unclassified repeats, satellites, simple repeats, and low complexity sequence.

**Figure 1:**
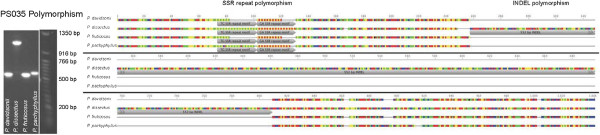
**An example of SSR and INDEL found in the comparisons of four *Penstemon *species in the sequences of marker Pen035.**

To assess the possibility of utilizing these markers in interspecific plant improvement studies, 12 of the 51 SSR/INDEL markers (Table 3) were tested on 93 mostly xeric *Penstemon* taxa (72 species [Table 1]) representing five of six subgenera recognized in the genus [14]. The overall success rate of the markers was 98% with 100% being polymorphic across the 93 taxa. Without sequencing each band and/or doing inheritance studies on each marker it is not possible to clearly determine if a polymorphism of a given marker is a variant of an allele or a new locus. However, we did amplify and sequence the amplicon produced at 11 of these markers in five *Penstemon* species (*P. cyananthus*, *P. davidsonii*, *P. dissectus*, *P. fruticosus*, and *P. pachyphyllus*). *P*. *pachyphyllus* var. *pachyphyllus* represents the largest subgenus (*Penstemon*) in the genus. These five species represented four of the presently classified six *Penstemon* subgenera. Of the 55 attempted sequences, 60% produced high quality sequences results which could be compared to the original 454 contigs containing the microsatellites. Using BLASTN (v2.2.25+) [41] we found that 33 sequences matched the respective microsatellite-containing contigs from which the SSR/INDEL markers were derived with an e-value of no more than 1.0e^-36^. An example of the types of polymorphism (SSRs and INDEL) found at these loci across the various species is represented graphically for the marker PS035 (Figure 1). For 22 (40%) of the 55 attempted sequences, we were unable to obtain high quality sequence information. In the majority of these cases (94%) the lack of high quality data was clearly due to the amplification of multiple amplicons (seen as multiple bands in gel electrophoresis) which impeded the sequencing of the PCR reaction. The source of the multiple amplicons may be from heterozygousity at the locus or from the amplification of paralogous loci.

Both the sequence data (Figure 1) as well as the marker size data (Tables 1 and 3) are clear evidence of sequence conservation, and probable homologous loci, in many of the SSR/INDEL markers. Marker PS012, the apparent most conserved marker, had six unique molecular weight bands and was present in all 93 taxa. The marker with the most diversity in its molecular weights was PS011 which had 18 variants and was not readable in seven of the 93 taxa. Of the 1,116 possible marker × taxa interactions, 22 (2.0%) did not produce reliable data. Seven of those 22 (0.5%) were absent of any product with the remaining 15 producing multiple bands (reported as ambiguous data). Clearly readable double bands were found in 135 of the 1,116 (12.1%) marker × taxa interactions (Table 1).

Our data suggest a high degree of sequence conservation across the genus, favoring the present hypothesis of a recent and rather rapid evolutionary radiation of the genus [13,14]. Furthermore, our data agree with Morgante et al. [45] who suggest that SSR presence in non-coding sequence are highly conserved and predate recent genome expansions of many plants. Some of our markers differed in length by as much as 570 bp (Tables 1 and 3) suggesting the presence of INDELs and possibly additional SSRs (Table 3). We confirmed the presence of INDELs in the sample of 11 markers which we sequenced (Figure 1). In some instances, these large fragment length variances may be amplifying a different locus, which is a recognized concern when using SSR based markers above the species level [46,47]. INDELs are useful as PCR based markers since they, like SSRs, are codominant and abundant in the genome and are commonly used in genetic mapping [26]. By combining the SSRs we identified in the source sequence for each of these markers with potential INDELs, alleles will be easily and inexpensively identified by gel electrophoresis.

To assess the possibility of phylogenetic (i.e., hierarchical) structure of the variation within these SSR/INDEL data at the broad taxonomic scale of our survey, we analyzed the 12 marker data set (Table 1) with PAUP*. Fast bootstrapping recovered a largely unresolved topology suggesting rampant homoplasy. Or one or more of these markers represent more than one locus. These results are similar to what others have reported about SSR type markers. SSRs have demonstrated utility for population and intraspecific relationships, such as cultivar differentiation; however, they can be problematic when used to reconstruct relationships above the species level where length differences are expected to poorly reflect homology [47,48]. Nonetheless, with over 96% of these SSR/INDEL regions being conserved across *Penstemon*, these markers have potential for studies of interspecific hybridization and cultivar development.

Interspecific *Penstemon* breeding is complex [7,11,15,49]; thus, having a set of inexpensive and easily used SSR/INDEL markers, which amplify across the genus, will have utility in understanding the results of some wide crosses. Empirical studies of various *Penstemon* interspecific crosses have ranged from a clearly recognizable intermediate phenotype of the two parents, to the F^1^ essentially mimicking one of the two parents, usually mirroring the female parent. Furthermore, in some instances the F^2^’s and additional generations continue to mimic the female parent to the point that Viehmeyer [49] began to question if apomixis was involved. An example of this phenomenon was a ‘Flathead Lake’ × *P. cobaea* interspecific cross. It was not until the hybrid progeny of this cross was crossed with other interspecific hybrids when the progeny gave a much wider range of phenotypes [49]. A probable reason for this phenomenon is “unequal segregation” which has been described in other wide crosses [50,51]. Thus through the use of these SSR/INDEL markers, regions of the genome can be identified which are unusual genotypic combinations, for that specific cross, and selections made accordingly [51-54]. Thus increasing the number of unique genotype/phenotype plants to be grown out to maturity from thousands of seedlings. Since many *Penstemon* require two years before their first anthesis, using markers to identify the greatest number of genotypic diverse plants is potentially very useful in the breeding of this crop.

**Figure 2:**
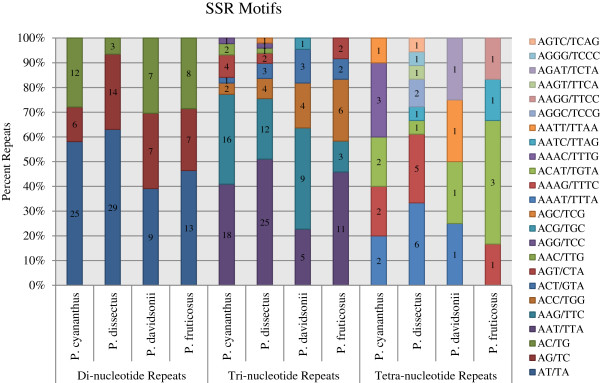
**Simple sequence repeat (SSR) motif distributions identified in each of the four *Penstemon *(*P. cyananthus*, *P. dissectus*, *P. davidsonii*, and *P. fruticosus*) sequences using the program MISA.**

Beyond amplification ability, we also assessed the composition and trends of all SSRs identified. On average, adenine and thymine rich repeat motifs were the most common repeat type in the di-, tri-, and tetra-nucleotide repeat motifs (Figure 2). In general, AT motifs are the most common motifs in noncoding regions of most plant genomes [45]. More variation was observed in the repeat motifs in the tetra-nucleotide repeats across the four species. Even closely related *P. fruticosus* and *P. davidsonii* had completely distinct tetra-nucleotide repeat motifs (Figure 2). This is likely due, in part, to the rarity of the motifs and high number of possible nucleotide combinations. Several studies have found that the hypothetical origins of some SSRs are retrotransposition events [48,55,56] and, as such, may be useful in developing part of a unique “fingerprint” for a given species.

### SNP analysis

Using our SNP discovery parameters of an 8× minimum coverage, and 30% representation of the minor allele, we identified an average of one SNP per 2,890 bp across the four species ranging from *P. cyananthus* (1/1,855 bp) to *P. fruticosus* (1/3,777 bp). The three species with similar genome sizes all had similar SNP frequencies (Table 6). As reported in other plant species [57,58], we found that the frequencies of bp transitions (A↔G or C↔T) were more common compared to transversions (A↔T, A↔C, G↔C, G↔T) in *Penstemon* by an average factor of 1.5 (Table 6). This is close to the 1.4 factor in *Arabidopsis*[35]. In the dual species assemblies, using the same parameters and a 90% SNP identity, the average transition to transversion mutation rate was lower at 1.2 (Table 6).

**Table 6:** SNP type and distributions along with SNP comparisons of sequences found within and between species (homologous sequence comparisons) using SNP_Finder_Plus (8X min. coverage, 30% min. minor allele, 90% min. identity)

**Species assembly**	**SNP**	**Average coverage**	**SNPs/assembly length**^**1**^	**SNP distribution**					
				**A↔C**	**A↔G**	**A↔T**	**C↔G**	**C↔T**	**G↔T**
*P. cyananthus*	2,493	16.4	0.000539 (~1/1855 bp)	10.7%	29.5%	13.9%	4.3%	30.2%	9.5%
*P. dissectus*	737	14.3	0.000280 (1/3568 bp)	9.8%	30.7%	15.6%	4.6%	27.4%	9.8%
*P. davidsonii*	713	14.4	0.000300 (~1/3333 bp)	11.9%	26.4%	15.2%	3.9%	28.3%	11.8%
*P. fruticosus*	615	12.4	0.000265 (~1/3777 bp)	11.7%	27.2%	17.9%	4.2%	25.4%	12.0%
Homologous sequence comparisons									
*P. cyananthus* × *P. dissectus*	3,253	10.6	0.009610 (~1/104 bp)	11.7%	27.5%	16.0%	7.1%	27.1%	10.6%
*P. cyananthus* × *P. davidsonii*	1,958	10.7	0.008062 (~1/124 bp)	11.1%	27.6%	15.8%	7.1%	28.5%	9.9%
*P. cyananthus* × *P. fruticosus*	2,015	10.6	0.008367 (~1/119 bp)	10.6%	27.2%	16.7%	6.8%	28.7%	10.1%
*P. dissectus* × *P. davidsonii*	2,348	10.8	0.015605 (~1/64 bp)	12.6%	26.7%	15.5%	7.5%	27.3%	10.4%
*P. dissectus* × *P. fruticosus*	2,133	10.0	0.011991 (~1/83 bp)	12.0%	26.4%	16.5%	7.6%	27.2%	10.4%
*P. davidsonii* × *P. fruticosus*	2,156	10.1	0.008404 (~1/119 bp)	12.8%	28.2%	14.5%	7.2%	27.2%	10.1%

^1^ Assembly length is bases shared between assemblies (see Table 4).

In the dual species assembly, we found an average of 1 SNP/97 bp between homologous sequence assemblies of any two of the four species. The frequency of SNPs between homologous sequences of *P. dissectus* and *P. davidsonii* was the highest at 1/64 bp, with the lowest being between *P. cyananthus* and *P. davidsonii* at 1/119 bp. These results are in line with previous molecular based studies [5,14]. *Penstemon davidsonii* and *P. fruticosus* both belong to subgenus *Dasanthera*, while *P. cyananthus* and either *P. davidsonii* or *P. fruticosus* homologous sequences had fewer SNPs at 1/124 and 1/119, respectively. All homologous sequence comparison involving *P. dissectus* had the highest density of SNPs (Table 6) suggesting that *P. dissectus* is the most evolutionary distant of the four species.

It is important, for a high degree of confidence in the results, when the “SNP identity” parameter in SNP_Finder_Plus to have two or more independent samples from the same species. This requirement was not met for each of the species assemblies, thus, introducing a weakness in our interspecific SNP comparisons. Although with the parameters of a minimum 8× coverage and minor allele frequency set at least 30%, a putative SNP must be present in at least three of the eight contig reads, thus providing some protection from mislabeling a sequencing and/or assembly error as a SNP. Furthermore, when doing across species comparisons the average SNP coverage was actually 14.4× (Table 6). Therefore, on average, five identical putative SNPs represented the minor allele.

To understand the viability of our interspecific SNP as markers, we utilized the 1,958 *P. davidsonii* × *P. cyananthus* and 2,348 *P. davidsonii* × *P. dissectus* SNPs identified in the 14,254 and 10,053 respective homologous contig parings (Tables 4 and 6). After removing contigs absent of identifiable SNPs, putative repetitive elements, and nonnuclear plastid DNA, 431 remained. Of these contigs, 99 were homologous across all three species (*P. cyananthus*, *P. davidsonii* and *P. dissectus*) another 164 were only in the *P. davidsonii* × *P. cyananthus* comparisons while the remaining 168 were in the *P. davidsonii* × *P. dissectus* contigs. Of those 431 contigs, we selected the first 192 for SNP marker development, 86 from each of the species comparisons. These contigs were utilized for competitive allele-specific PCR SNP primer design using PrimerPicker (KBioscience Ltd., Hoddesdon, UK).

Of the 192 SNP markers tested, using KASPar genotyping chemistry, 75 (39%) of produced consistent results for *P. cyananthus*, *P. davidsonii*, *P. dissectus*, and *P. fruticosus* (Table 7). All 75 SNP markers indicated polymorphisms between *P. cyananthus*, *P. davidsonii*, and *P. dissectus*, where only 16 (21% of the 75) produced results in *P. fruticosus* (Table 7). These results suggest that it is possible to develop intrageneric SNPs for *Penstemon*. However, it is unclear as to how viable these markers will be for use across all the species of the genome since only 21% worked on all the species used in this GR-RSC study.

**Table 7:** ***Penstemon *SNP marker name, GenBank dbSNP accession ID, polymorphism type, KASPar™ primer sequences (A1, A2 and common allele specific reverse) for all 75 functional SNP assays**

**Name**	**Contig Source**^**1**^	**SNP Allele GenBank Accession #**^**2**^	**SNP Type**	**Allele Specific A1 Forward (5′→3′) **^**3**^	**Common Allele Specific Reverse 5′→3′)**	***P. davidsonii***	***P. cyananthus***	***P. dissectus***	***P. fruticosus***	***P. pachyphyllus***	***P. cyananthus + P. davidsonii***	***P. dissectus + P. davidsonii***
				**Allele Specific A2 Forward (5′→3′) **^**3**^								
PenSNP00001	00336CD	JX649978	A/G	AAGATTGCATGGAGAGGAAATGGATT	CGATCCAAATGGCAGATCCGAGAAA	X^4^	Y	X		X	Y	Y
				AGATTGCATGGAGAGGAAATGGATC								
PenSNP00002	00405CD	JX649979	C/T	ACGCGAGTAATAAGTTGGTTTTCTTC	CCAACACTTCCGCAGAAGCTCTTAA	Y	X	Y	X	Y	H	H
				GACGCGAGTAATAAGTTGGTTTTCTTT								
PenSNP00003	02625CD	JX649980	A/T	AAAAGCTCCCAAACATGACTATGAACT	AATTCTTCGACACTTGAAGAGAGCGTAA	Y	X	Y		Y	H	H
				AAAAGCTCCCAAACATGACTATGAACA								
PenSNP00004	02857CD	JX649981	A/C	ATCAAATGAACTTGTCTCATGAGCCT	GCAACAAGGTGCAAAAAATTGTAGCGTAA	X	Y	X		X	H	H
				CAAATGAACTTGTCTCATGAGCCG								
PenSNP00005	03943CD	JX649982	A/G	ACTACCAAAACTACCCTTCCCTTA	GGGGTACAGAGTTGAGAAGAAGGAA	X	Y	X		X	H	H
				ACTACCAAAACTACCCTTCCCTTG								
PenSNP00006	04420CD	JX649983	A/C	TGTCTCTAAATCGATATGATGAGGCT	GTGGTTCTTCCCCTTTAGAGGACTT	Y	X	Y	X	Y	H	H
				GTCTCTAAATCGATATGATGAGGCG								
PenSNP00007	08446CD	JX649984	A/T	GGCAACATCCTCAGCAGAGACA	CCGACTCCCTTAGCAATCTTAGCAT	Y	X	X		X	H	X
				GGCAACATCCTCAGCAGAGACT								
PenSNP00008	11303CD	JX649985	C/G	GGGTGGTATTGGTTACTTTTATGGG	CGGTATAAGAGCAACTAAGCTAAATGACTT	Y	X	Y		Y	H	H
				GGGTGGTATTGGTTACTTTTATGGC								
PenSNP00009	11357CD	JX649986	C/T	ACAATATTTGATAATTCATTCTCAAGTGCG	AAGCATGCAGTGAGACAAAAGCTAAGAT	X	Y	X	Y	X	H	H
				CACAATATTTGATAATTCATTCTCAAGTGCA								
PenSNP00010	11935CD	JX649987	A/C	AGCCTGATTATCCCTTAAACCCAATT	GAATCACGGCGGGGGAGCAAAT	X	Y	X		X	Y	
				GCCTGATTATCCCTTAAACCCAATG								
PenSNP00011	12047CD	JX649988	C/T	TTTGGCACTGCAGTGACCATC	TGCTCCAGTCCGAAGGAAGTTGAAT	X	Y	Y		X	Y	Y
				CTTTTGGCACTGCAGTGACCATT								
PenSNP00012	12119CD	JX649989	A/G	AAGATAGACGTGGTATTTCTTCAGCA	GCAATTAGTCACAGACCATAGTGG	X	Y	X		X	H	H
				AGATAGACGTGGTATTTCTTCAGCG								
PenSNP00013	12398CD	JX649990	A/T	TATTTTCCTTTCTGCAATCTCAACATTGA	GTTGAGTGTGATTTTAGAGTGCATTTAGTT	X	Y	X		X	Y	Y
				ATTTTCCTTTCTGCAATCTCAACATTGT								
PenSNP00014	13398CD	JX649991	A/C	AGGCCTGTGGCTGACTTGTCA	GGCATATCTTTGCCCGTTTCCACAA	X	Y	X	X	X	H	H
				GGCCTGTGGCTGACTTGTCC								
PenSNP00015	13752CD	JX649992	A/C	AAATGCTCCCTCATTTTGACCATATGA	GTCAACGGATTTGTGGAAGTCGGTA	Y	X	Y		Y	H	H
				ATGCTCCCTCATTTTGACCATATGC								
PenSNP00016	14394CD	JX649993	C/G	TGAAAATTTCAGATTTAATGAACAAACAGTC	AGACTTGTAACAAATTCCTTGGGTCCAAA	X	Y	X			Y	H
				GAAAATTTCAGATTTAATGAACAAACAGTG								
PenSNP00017	14661CD	JX649994	A/G	TGACCAAGGAATCTGTTCAAGAACTT	CTTCTACTGTGGCTGTTTCACCTCTA	Y	X	Y			H	H
				GACCAAGGAATCTGTTCAAGAACTC								
PenSNP00018	15226CD	JX649995	G/T	TACCTCCAATTGTGATGCAACATTAG	CTAAGTGAGAAGCACAAGGA	X	Y	X		X	H	H
				CTTACCTCCAATTGTGATGCAACATTAT								
PenSNP00019	17421CD	JX649996	G/T	ATCCTCCTCCTTTGCATCAAAGC	GAGCCAACCTCGACTGCTTCTATTT	Y	X	Y		Y	X	H
				CATCCTCCTCCTTTGCATCAAAGA								
PenSNP00020	17816CD	JX649997	A/G	AAGGACTGAGTACCAAGACAGATCT	GCCAGGGTACTGAACCTGTCTTTTA	X	Y	X		X	H	H
				GGACTGAGTACCAAGACAGATCC								
PenSNP00021	18745CD	JX649998	C/T	AGCATATTGAAAAGATCAGTCGCATAG	CAGCTGCTCCTATCCAATCTTCGAA	Y	X	Y		Y	H	H
				AAAGCATATTGAAAAGATCAGTCGCATAA								
PenSNP00022	19267CD	JX649999	A/G	AAATACCTGAGCTTCTGCCTCTTGT	GATGCTCGTCATCTTGCTCAACGAT	X	Y	X	Y	X	H	H
				ACCTGAGCTTCTGCCTCTTGC								
PenSNP00023	21409CD	JX650000	C/G	ACCATTCAGGTAATATTTCCAAAGGC	AGCGGTTCTAGAACCGTCAATGCTT	Y	X	Y	Y	Y	X	H
				ACCATTCAGGTAATATTTCCAAAGGG								
PenSNP00024	22934CD	JX650001	A/G	GTACAATTGTCAAGTGTGTATTTTCTTACATA	GCACTGCACCATTCATGCCCTAAAA	Y	X	Y		Y	H	H
				ACAATTGTCAAGTGTGTATTTTCTTACATG								
PenSNP00025	22942CD	JX650002	A/T	ATCCGATTCTTCGTCTACTATGCCA	AGAAAAGCACAAGCTGAAATCAGGGAA	X	Y	X		Y	H	H
				ATCCGATTCTTCGTCTACTATGCCT								
PenSNP00026	27992CD	JX650003	A/G	TCCTCCTCGTCTCTTCCTCTT	CTTGGACCGTCCAAAGAAGGAAAGAA	Y	X	Y		Y	H	H
				CCTCCTCGTCTCTTCCTCTC								
PenSNP00027	01179DD	JX650004	A/G	TCGACCCCAACCTGTCACA	CTTGCTTGGTTTCGGAAAGAG	Y	X	Y		Y	H	H
				CTTCGACCCCAACCTGTCACG								
PenSNP00028	01235DD	JX650005	C/T	TGTGATCTTTGGTTTGAACTTTGTC	CTACCAAACTCACTCTAACATCCGGAT	X	Y	X		X	H	H
				CACTTGTGATCTTTGGTTTGAACTTTGTT								
PenSNP00029	01600DD	JX650006	A/G	TGGTCTTGTTCTTTACCATTACGCAT	GAAGTAGCTGCCATGGAAAAGGAAGTT	X	X	Y	X	X	X	H
				GGTCTTGTTCTTTACCATTACGCAC								
PenSNP00030	04630DD	JX650007	A/G	AGTAGTACAGAATACTTAAAACTATCACCA	GTTGGGGGAGTTGCCTTCTTGAAAT	X	X	Y		X	X	H
				GTAGTACAGAATACTTAAAACTATCACCG								
PenSNP00031	05304DD	JX650008	C/T	AGTTTTCCTTTTGTCCTTATGTGCAG	AAGGCTTAGCTTGGATGATATCCTACAA	Y	Y	X		Y	Y	Y
				CAGTTTTCCTTTTGTCCTTATGTGCAA								
PenSNP00032	05884DD	JX650009	A/T	GTCACCGCCTCCGATTGAGATT	CGGCTTTTGACGCCGCCGTAAA	X	Y	X		X	H	H
				GTCACCGCCTCCGATTGAGATA								
PenSNP00033	06956DD	JX650010	G/T	GTTGATTCTACAGATCTTAATTCTTGATTG	TACTACAAAGGGTAAAAAGTGCAATTCATA	X	Y	X		X	H	H
				AGTTGATTCTACAGATCTTAATTCTTGATTT								
PenSNP00034	08307DD	JX650011	C/G	ACATTAAGGGTCCACCAAAAATCCG	GCGCAATTAAAATCTCTTAAATCACCTGGT	Y	Y	X			Y	H
				ACATTAAGGGTCCACCAAAAATCCC								
PenSNP00035	08352DD	JX650012	A/T	AGTACAAGGAAAAACCTTTTATTAGTAAGTATA	CTGACACAAACCCATTCTAATATGACCAA	X	Y	X		X	H	H
				AGTACAAGGAAAAACCTTTTATTAGTAAGTATT								
PenSNP00036	08488DD	JX650013	A/T	GTGTTGGAGAGCCAGGTGCGA	GTATTGAGGATCATTCTGACAAAAAACATA	Y	X	Y		Y	H	H
				GTGTTGGAGAGCCAGGTGCGT								
PenSNP00037	08608DD	JX650014	C/T	GTAGATAAGTTGATTGCGAGAGGC	CCAAACAAATGCACCACATTCTCCTT	X	Y	X	Y	X	Y	H
				GGTAGATAAGTTGATTGCGAGAGGT								
PenSNP00038	08831DD	JX650015	A/T	TTTGAACTGCCATGTAAAGTTGTTTTAGA	ATTTTGAACCAAGGAGCTATCAGAGG	X	Y	X		X	Y	H
				TTGAACTGCCATGTAAAGTTGTTTTAGT								
PenSNP00039	08947DD	JX650016	A/T	GGGATCGTAAAACTCAGGAAAAATGA	TCAGATACTCGTGGGGTCTTCGATT	X	Y	X	H	X	Y	H
				GGGATCGTAAAACTCAGGAAAAATGT								
PenSNP00040	08959DD	JX650017	A/G	AGAGAATGAAGAAGGAGAAGGAAGAAA	CTCCTACGGTTGCATTATCGGTAGTA	Y	X	Y		Y	Y	Y
				GAGAATGAAGAAGGAGAAGGAAGAAG								
PenSNP00041	09272DD	JX650018	A/T	TTCTACAAAACAATCAGCAGTCATCATT	TCGACACCTTTTGCCTTATCTTGAA	X	Y	X		X	Y	H
				TCTACAAAACAATCAGCAGTCATCATA								
PenSNP00042	09369DD	JX650019	C/T	GTTTTTATACGCATCCATATACATAATAATAG	GGTTCACTCTCCAGAAATAAAATCTTATAT	Y	X	Y		Y	H	X
				GTTTTTATACGCATCCATATACATAATAATAA								
PenSNP00043	09764DD	JX650020	A/G	AATTCAACGTCAAATTGCAAGGTTGCA	TTCACTATACCGGCTGAGTTGGCAT	Y	X	Y		Y	H	H
				CAACGTCAAATTGCAAGGTTGCG								
PenSNP00044	10765DD	JX650021	A/G	TTTTTTAATAAATATCCTGGTGGATAATTTAT	AAATTGAGTGGATGGCTAGGAAGACTAA	X	Y	X	Y	X	H	H
				TTTTTAATAAATATCCTGGTGGATAATTTAC								
PenSNP00045	10870DD	JX650022	A/T	AGATCTGGAGACTAAAT	CGAAGAGTTTGGGTGGGCGGAT	X	Y	X		X	Y	Y
				AGATCTGGAGACTAAAA								
PenSNP00046	11107DD	JX650023	C/T	GTCCGACGTGACAATGCAGC	CGCCGTCAAAGAGACTTTGTTGGAT	Y	X	Y		Y	H	H
				CTGTCCGACGTGACAATGCAGT								
PenSNP00047	11531DD	JX650024	C/T	AGAAGATTCTTCGGCTGGGAGC	TCTTCACATGATTACGACAATGGCTGAAT	X	Y	X		X	H	H
				AAGAAGATTCTTCGGCTGGGAGT								
PenSNP00048	11655DD	JX650025	A/G	ACGTCCATGGAGGACCATAAA	GCTGTCTTCCTGCAAGGAACTTCTT	X	Y	X		X	H	H
				CTACGTCCATGGAGGACCATAAG								
PenSNP00049	11974DD	JX650026	G/T	AAAATGCATGTAGTTTGGTTTACG	CACACCCCCAAAGGAAGAATAGCAT	Y	X	Y		Y	H	H
				AAAATGCATGTAGTTTGGTTTACT								
PenSNP00050	13159DD	JX650027	C/T	TGAATGTACTTTTCATTGATAGAGAACG	AACAATAGTACAACACAACTAAAGCAGAGA	Y	Y	X		Y	Y	H
				GTTGAATGTACTTTTCATTGATAGAGAACA								
PenSNP00051	13463DD	JX650028	G/T	GCCTTTGACGGCGAAGGATTTC	GCAAGCACGGCACTAAGCCCTT	X	Y	X		X	H	H
				CGCCTTTGACGGCGAAGGATTTA								
PenSNP00052	14334DD	JX650029	A/G	AGAAACAACAAATACGAATAAATCACCCA	TTCGAAAATTGTGCTTGAATCACGCAGT	X	X	Y	H	X	X	Y
				GAAACAACAAATACGAATAAATCACCCG								
PenSNP00053	00290DD03373CD	JX650030	C/T	TGCCTTTGCGTCGCCACAATC	AGCTAAGAGATGGGCAGACTTTACAAAAT	Y	X	Y		Y	H	H
				CTTGCCTTTGCGTCGCCACAATT								
PenSNP00054	00354DD04637CD	JX650031	A/G	GCAAAAGGGAACCCTCATTTCGTT	TACTTGTCTGGGACTTTTCCTTTCTCTTT	X	X	Y		X	X	H
				CAAAAGGGAACCCTCATTTCGTC								
PenSNP00055	01161DD11697CD	JX650032	A/G	ACTGGTAAATACACTACGTTCACAGT	GAAACACAGCAGCCCAACGACATAT	Y	Y	X	X	Y	Y	H
				CTGGTAAATACACTACGTTCACAGC								
PenSNP00056	01323DD15501CD	JX650033	A/G	ACCTGAAGAATTTGTTCACTACTTCGT	GGATCGGGTGGAACGATTTGTGTT	X	Y	X		X	H	H
				CCTGAAGAATTTGTTCACTACTTCGC								
PenSNP00057	01541DD02481CD	JX650034	A/G	AATTAGAACCACATCCACTGATTCCAA	GGAGCCCAAACCTTTTACATTCTTTTCTA	Y	X	Y		Y	H	H
				AGAACCACATCCACTGATTCCAG								
PenSNP00058	02019DD03127CD	JX650035	A/G	GTGATTGTTAAATCTGAATATATAATTTCTTTT	GTACGAGGCTTCGAAAAAGACCAGAT	X	Y	X		X	H	H
				GTGATTGTTAAATCTGAATATATAATTTCTTTC								
PenSNP00059	02851DD17191CD	JX650036	A/G	AAGAGGTTGATCCTAAGTTATCGAGA	GAAGAAAATCATTGTCCACATCTCGTGTA	X	Y	X		X	Y	H
				AGAGGTTGATCCTAAGTTATCGAGG								
PenSNP00060	03089DD14703CD	JX650037	C/T	TTTCAGAGTCACTAATGTTCTCACG	GCATTTCTTGTCCATCTCTTCAAGATGTA	X	X	Y	Y	X	X	H
				GTTTTCAGAGTCACTAATGTTCTCACA								
PenSNP00061	03423DD25897CD	JX650038	A/C	AATTCTTCTACGTCCATTTGATCGGAT	TATTCTTAGACATGGACATGGAAATTGAGA	Y	X	Y		Y	H	H
				CTTCTACGTCCATTTGATCGGAG								
PenSNP00062	04632DD19186CD	JX650039	A/T	AAATGGGTCAGCTGAAATTTCCGCA	CTCTTCTTTACTCTGTTTTTTCTTCTTTTT	Y	Y	X			Y	H
				AAATGGGTCAGCTGAAATTTCCGCT								
PenSNP00063	05160DD08243CD	JX650040	C/G	TCGATCGTTGAAATGATAATTGATACAAG	GATCCCATAGACTTCTTTTAAGGATTCTAA	Y	X	Y	Y	Y	H	H
				CGATCGTTGAAATGATAATTGATACAAC								
PenSNP00064	06332DD03627CD	JX650041	A/C	ATCAAATGCCATAGATCCTGCAGATTT	ACATTTCCTACACCAACTTCTTCCACTA	X	X	Y		X	X	H
				CAAATGCCATAGATCCTGCAGATTG								
PenSNP00065	08748DD13630CD	JX650042	C/G	AGCTGTTCAGGAGGTTCATGAATG	CACCATGTGAACCAACACTATTGTCATTT	X	Y	X		X	H	H
				AGCTGTTCAGGAGGTTCATGAATC								
PenSNP00066	09773DD14323CD	JX650043	A/G	TCATGCCCATTCCCCA	CCTGGTATGAACATGGGGAGGTTAT	Y	Y	X		Y	Y	
				CATGCCCATTCCCCG								
PenSNP00067	10248DD06150CD	JX650044	C/G	TGTGTCATTGAAATCAATCCGC	GTTTCATATCTCCCTTTGAGCTTCTTGAA	Y	Y	X	Y	Y	Y	H
				GCTTGTGTCATTGAAATCAATCCGG								
PenSNP00068	10624DD11358CD	JX650045	A/G	GTGGCAGTGTGAAACTGCATCA	GTTTTTCCCTGGGTGCTAAGGTTCAT	Y	X	Y		Y	H	H
				GTGGCAGTGTGAAACTGCATCG								
PenSNP00069	11267DD06273CD	JX650046	A/C	ACCAAATACTTATTAGCTCCAGTCGAA	GACTGAAGGATGTTGCGAGAGGC	Y	Y	X		Y	Y	H
				CCAAATACTTATTAGCTCCAGTCGAC								
PenSNP00070	11564DD17128CD	JX650047	C/T	TGGACTTGGCATTGAAACAAAAGATC	ATATGAAACTCCCCACAAGAAA	Y	X	Y		X	H	H
				AATTGGACTTGGCATTGAAACAAAAGATT								
PenSNP00071	11647DD17264CD	JX650048	C/G	GCACGAGCCAAAATCCTGAGC	ATTGGCATGTGTATCCTGTGTGGGA	X	Y	X	Y	X	H	H
				GCACGAGCCAAAATCCTGAGG								
PenSNP00072	11671DD17144CD	JX650049	C/T	GTGCAGCAACCCCTATTCATGAC	CCTGTCCAAAACATATGATCTTCATTGGAA	X	Y	X			H	H
				ATGTGCAGCAACCCCTATTCATGAT								
PenSNP00073	12915DD17470CD	JX650050	A/G	AAGAAAAGGGTGGACAAATTAAACCGT	CAGAACAACATCATACTTGATAAATCTCTT	X	X	Y		X	X	H
				GAAAAGGGTGGACAAATTAAACCGC								
PenSNP00074	13828DD14937CD	JX650051	C/T	GTAAGATATGCTGCCAGATGG	CTCTGAAGAAGTTTTTGTCCTTGATAGCTA	Y	Y	X		Y	Y	H
				GTAAGATATGCTGCCAGATGA								
PenSNP00075	14286DD18608CD	JX650052	G/T	GTATTGAGAGCCACTACCGG	CCACTTGAATTGTTTGAAGAGTTTGGGAA	Y	X	Y		Y	X	H
				CTGTATTGAGAGCCACTACCGT								

^1^These contigs have been deposited at DDBJ/EMBL/GenBank as a Whole Genome Shotgun project under the accessions AKKG00000000 (*P. cyananthus*), AKKH00000000 (*P. dissectus*), AKKI00000000 (*P. davidsonii*), and AKKJ00000000 (*P. fruticosus*). The version described in this paper is the first version for each accession, XXXX01000000.

^2^The GenBank accession identification for the full sequence for each allele with the specific SNP bp identified.

^3^KASPar™ primers: A1 and A2 primers are SNP allele specific. All A1 Forward primers had the follow universal primer GAAGGTGACCAAGTTCATGCT added to the 5′ end of the allele specific sequence. All A2 Forward primers had the follow universal primer GAAGGTCGGAGTCAACGGATT added to end of the 5′ allele specific sequence.

^4^H = heterozygous compared to either homozygous condition for either “X” or “Y”.

### Repetitive elements

**Figure 3:**
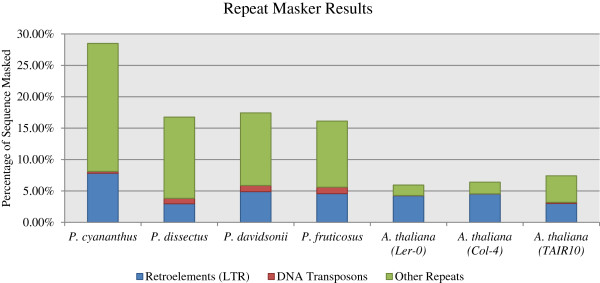
**Percentage of retroelements, DNA transposons and other unclassified repeats in *Penstemon cyananthus*, *P. dissectus*, *P. davidsonii*, *P. fruticosus*, and both genome reduced and non-genome reduced *Arabidopsis***^**1**^**. **^1^ Genome reduced *A. thaliana* sequence from Maughan et al. [35]; *A. thaliana* RILs Ler-0 and Col-4; Non-genome reduced *A. thaliana* sequence downloaded from TAIR (The *Arabidopsis* Information Resource) as whole chromosomes; the diploid (2n = 2x = 16) genome size as reported by Broderick et al. and Schmuths et al. [5,59].

**Figure 4:**
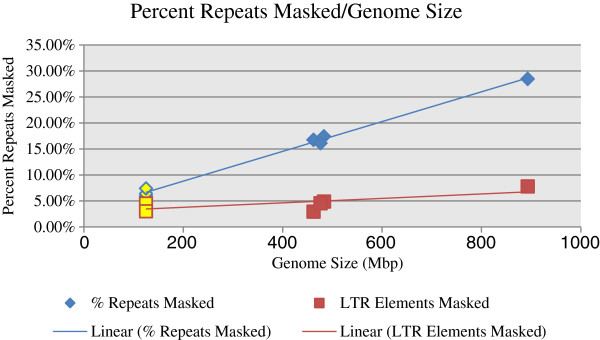
**Relationship between genome size and repeat elements in *Penstemon *including the relationship of both LTRs and total repeat elements to genome size for both genome reduced *Penstemon *and genome reduced/non-genome reduced *Arabidopsis***^**1 **^**(yellow). **^1^ Genome reduced *A. thaliana* sequence from Maughan et al. [35]; *A. thaliana* RILs Ler-0 and Col-4; Non-genome reduced *A. thaliana* sequence downloaded from TAIR (The *Arabidopsis* Information Resource) as whole chromosomes; Genome size as reported by Broderick et al. and Schmuths et al. [5,59].

We identified 28.5%, 16.8%, 17.4% and 16.1% of the respective sequence from *P. cyananthus*, *P. dissectus*, *P. davidsonii*, and *P. fruticosus* as repeat elements using RepeatModeler and RepeatMasker. Of these elements, 3.0-7.8% were identified as LTR (long terminal repeat) retroelements, 0.3-1.0% transposons and the remainder were unclassified (Table 5). Since RepeatModeler utilizes RECON and RepeatScout to create a de novo model in RepeatMasker in place of the *Arabidopsis* model, details about the subcategories of LTRs and transposons which are included in the model could not be addressed. Maughan et al. [35] utilized GR-RSC on the *Arabidopsis* lines Ler-0 and Col-4. Utilizing RepeatModeler, then RepeatMasker on their sequence data from these lines, we found an average of 6.2% were identified as repetitive elements, of which 4.4% were identified as LTR retroelements and 0.4% were transposons. By way of comparison, the downloaded full “non-genome reduced” sequence of *Arabidopsis* line TAIR10 had a similar 7.4% of the sequence identified as repeat elements of which 3.0% were LTR retroelements and 0.2% were transposons (Table 5 and Figures 3 and 4). These data suggest that the GR-RSC method reflects, at least for repetitive elements, similar proportions as to that found in the full sequence of *Arabidopsis*.

Broderick et al. [5] hypothesized that the broad range found in *Penstemon* genome sizes, of the same ploidy, may be explained by retrotransposons. Lynch [60] detailed a relationship between genome size and repeat elements suggesting a linear relationship between the number of elements and genomes size [60-62]. The four *Penstemon* species used in this study provide insufficient evidence to establish a linear relationship between genome size and repeat elements in *Penstemon*. However, the three smaller, similar sized, *Penstemon* genomes possess comparable quantities of repetitive elements whereas *P. cyananthus* (the largest genome) has nearly double the number of repeat elements compared to the other three species (Figure 3).

Not only do repetitive elements largely influence genome size, but they are also likely to evolve more rapidly than do low-copy sequence [62,63]. Thus, repetitive elements of a species take on unique “fingerprints” which become valuable in phylogenetic relationship studies [64,65]. Thus, our limited four *Penstemon* species genomic data set suggest agreement with the two hypotheses that firstly, repetitive elements are a major component of the genome size variation identified by Broderick et al. [5]. Secondly, these elements are variable between the species we tested suggesting the possibility of identifying species specific repetitive elements. However, without further comparisons we were unable to identify specific repetitive elements associated with the four *Penstemon* species used in this study.

### Gene ontology

Using BLASTX we identified an average of 21.5% of the contigs across the four species as putative genes with an average of 13.9% annotated by Blast2GO (Table 5). These putative genes were compared and contrasted in a more detailed study by Dockter [23]. Furthermore, he compared the *Penstemon* sequences to known genes from the related genera *Antirrhinum* and *Mimulus*, and identified nine putative *Penstemon* genes from *Antirrhinum* and 14 from *Mimulus* with an e-value below 1.0e^-13^. Three genes (NADH dehydrogenase from *M. aurantiacus*, ribosomal protein L10 from *M. guttatus*, and ribosomal protein subunit 2 from *M. aurantiacus*, *M. szechuanensis*, and *M. tenellus var. tenellus*) were perfect hits (e-value = 0.0).

## Conclusions

*Penstemon* are recognized for their phenotypic variation and their adaptation to multiple environments [6-8,13,14,17,30,31]. Broderick et al. [5] found that this diversity is reflected by a wide range in their genome sizes. Nevertheless, even with this demonstrated plasticity we have identified evidence that there is a high level of sequence conservation across the genus. This apparent sequence conservation is in harmony with the hypothesis that *Penstemon* has rapidly irradiated to its variety of species rather recently in evolutionary time [13,14]. Furthermore, our study identified evidence that the genome size variation in *Penstemon* is rooted in the amount of repetitive elements in each species.

Despite the large differences in *Penstemon’s* genome size, the finding that the genus has a great deal of sequence conservation is invaluable for the development of interspecific markers. The further development and mapping of a number of conserved markers will facilitate the domestication of xeric *Penstemon* cultivars via interspecific hybridization which are largely unexploited largely due to crossing barriers [6-8,10-12,15]. Viehmeyer [16] hypothesized that it might be possible to develop *Penstemon* breeding lines that would facilitate the indirect interspecific hybridization of any two species within the genus. He and others have used traditional breeding techniques to develop a number of interspecific hybrids [7,11,15,17,66]. Clarifying the phylogenetic relationships within the genus should facilitate these objectives [67]. In the largest *Penstemon* phylogenetic study conducted to date, Wolfe et al. [14] sequenced the ITS and two chloroplast genes in 163 species. They concluded that many species are polyphyletic in their origins thus making them difficult to discriminate between one another; thus, requiring additional molecular studies to more accurately define taxonomic relationships.

We tested 51 SSR/INDEL based markers (Table 3), and identified several thousand inter- and intraspecific SNPs (Table 6), all of which have potential as both inter- and intraspecific markers. Of the 51 SSRs/INDELs we selected 12 to test across 93 *Penstemon* taxa. The resulting data was used to more clearly define the phylogenetic relationships of those taxa but our results were incoherent. It is possible that some of these markers may represent more than one locus in the *Penstemon* genome. This situation has been identified by others as a potential weakness in using SSR based markers in interspecific phylogenetic studies [46,47]. A major reason for the vagary in *Penstemon’s* phylogeny is that it appears to have quite recently evolved and rapidly radiated leaving weak species boundaries [13,14]. Furthermore, there are a number of reports of speciation via natural interspecific hybridization found within the genus [14,68-73]. Therefore, like Wolfe et al. [14], we concluded that better marker data sets will be required to reduce present phylogenetic ambiguity.

To gain clearer insights into the relationships of *Penstemon* it will take carefully designed large scale sequencing studies. There are methods which are showing promise to do such studies economically. One example would be to utilize GR-RSC or similar methods which will sample large quantities of homologous sequence of a genome at ever decreasing costs [18,20,74]. Since our SSR/INDEL, sequence, and SNP data have demonstrated broad applicability across *Penstemon* it becomes evident that further studies utilizing this same GR-RSC protocol and downstream analysis on additional species would allow broader comparisons of putative genes, repeat elements, SNPs and SSRs, facilitating a much better understanding of the genus. Furthermore, using this technique on carefully selected parents and their segregating progeny would allow *Penstemon* genetic mapping studies which would greatly enhance the ability to do breeding and domestication studies within the genus. Historically, studies of this nature would have been unthinkable; however, mass homologous loci sequence studies are rapidly becoming feasible [18,20,74]. In the interim it is possible to take the data we report here and further test the 75 SNPs we have reported here along with others not yet developed and for around US$0.05/data point [18,20] do a much broader study. Studies on homologous SNPs across many *Penstemon* taxa, similar to the *Amaranthus* study of Maughan et al. [20], should assist in developing improved insights into *Penstemon* phylogenetic relationships and produce high quality genetic maps from carefully designed segregating *Penstemon* populations.

## Competing interests

The authors declare no competing interests.

## Authors’ contributions

Rhyan B Dockter, David B Elzinga, Brad Geary, P Jeff Maughan, Leigh A Johnson, Danika Tumbleson, JanaLynn Franke, Keri Dockter, and Mikel R Stevens. RBD preformed the GR-RSC technique and either carried out or oversaw the all other steps of the study and participated in all planning and design of all experiments as well as their analysis and did the initial drafting of the manuscript. DBE did or assisted in all bioinfomatics performed in this study. BG participated in the design of all aspects of the study as well as advised RBD and was involved in the editing and revising of the manuscript. PJM advised and assisted in the GR-RSC technique as well as advised RBD in relevant issues of the bioinfomatics of the study and was involved in the editing and revising of the manuscript. LAJ advised and assisted RBD and MRS in the taxonomy related issues of the study and was involved in the editing and revising of the manuscript. DT, JF, and KD carried out all aspects, including basic analysis, of the marker studies reported. MRS was the senior advisor of RBD and was intricately involved in all aspects of the study and the manuscript. All authors both read and approved the final manuscript.

## References

[B1] St Hilaire R Arnold MA Wilkerson DC Devitt DA Hurd BH Lesikar BJ Lohr VI Martin CA McDonald GV Morris RL Pittenger DR Shaw DA Zoldoske DF Efficient water use in residential urban landscapes HortScience 2008 43 2081 2092

[B2] Martin CA Landscape water use in Phoenix, Arizona Desert Plants 2001 17 26 31

[B3] Bradley BA Blumenthal DM Early R Grosholz ED Lawler JJ Miller LP Sorte CJB D’Antonio CM Diez JM Dukes JS Ibanez I Olden JD Global change, global trade, and the next wave of plant invasions Front Ecol Environ 2012 10 20 28 10.1890/110145

[B4] Burt JW Muir AA Piovia-Scott J Veblen KE Chang AL Grossman JD Weiskel HW Preventing horticultural introductions of invasive plants: potential efficacy of voluntary initiatives Biol Invasions 2007 9 909 923 10.1007/s10530-007-9090-4

[B5] Broderick SR Stevens MR Geary B Love SL Jellen EN Dockter RB Daley SL Lindgren DT A survey of *Penstemon’s* genome size Genome 2011 54 160 173 10.1139/G10-106 21326372

[B6] Lindgren D Wilde E Growing Penstemons: Species, Cultivars and Hybrids 2003 Haverford, PA: Infinity Publishing Com

[B7] Lindgren DT Callaway DJ, Callaway MB Breeding *Penstemon* Breeding Ornamental Plants 2000 Portland, Oregon: Timber Press 196 212

[B8] Nold R Penstemons 1999 Portland, Oregon: Timber Press

[B9] Viehmeyer G Let’s breed better *Penstemon* Bul Amer Penstemon Soc 1955 14 275 288

[B10] Way D James P The Gardener’s Guide to Growing Penstemon 1998 Portland, OR: Timber Press

[B11] Lindgren DT Schaaf DM *Penstemon*: a summary of interspecific crosses HortScience 2007 42 494 498

[B12] Lindgren D List and Description of Named Cultivars in the Genus Penstemon (2006) 2006 Lincoln, Nebraska: University of Nebraska-Lincoln Extension; EC1255

[B13] Straw RM A redefinition of *Penstemon* (Scrophulariaceae) Brittonia 1966 18 80 95 10.2307/2805112

[B14] Wolfe AD Randle CP Datwyler SL Morawetz JJ Arguedas N Diaz J Phylogeny, taxonomic affinities, and biogeography of *Penstemon* (Plantaginaceae) based on ITS and cpDNA sequence data Amer J Bot 2006 93 1699 1713 10.3732/ajb.93.11.1699 21642115

[B15] Uhlinger RD Viehmeyer G Penstemon in your Garden 1971 Lincoln, Nebraska: University of Nebraska College of Agriculture The Agricultural Experiment Station Station Circular 105

[B16] Viehmeyer G Reversal of evolution in the genus *Penstemon* Am Nat 1958 92 129 137 10.1086/282021

[B17] Viehmeyer G Advances in *Penstemon* breeding Bul Amer Penstemon Soc 1973 32 16 21

[B18] Cronn R Knaus BJ Liston A Maughan PJ Parks M Syring JV Udall J Targeted enrichment strategies for next-generation plant biology Amer J Bot 2012 99 291 311 10.3732/ajb.1100356 22312117

[B19] Heslop-Harrison JS Exploiting novel germplasm Aust J Agric Res 2002 53 873 879 10.1071/AR02078

[B20] Maughan PJ Smith SM Fairbanks DJ Jellen EN Development, characterization, and linkage mapping of single nucleotide polymorphisms in the grain amaranths (*Amaranthus* sp.) Plant Gen 2011 4 1 10 10.3835/plantgenome2011.12.0001

[B21] Bernardo R Molecular markers and selection for complex traits in plants: learning from the last 20 years Crop Sci 2008 48 1649 1664 10.2135/cropsci2008.03.0131

[B22] Tanksley SD McCouch SR Seed banks and molecular maps: unlocking genetic potential from the wild Science 1997 277 1063 1066 10.1126/science.277.5329.1063 9262467

[B23] Dockter RB Genome snapshot and molecular marker development in Penstemon (Plantaginaceae). M.S. Thesis 2011 Brigham Young University, Department of Plant and Wildlife Sciences

[B24] Santana QC Coetzee MPA Steenkamp ET Mlonyeni OX Hammond GNA Wingfield MJ Wingfield BD Microsatellite discovery by deep sequencing of enriched genomic libraries Biotechniques 2009 46 217 223 10.2144/000113085 19317665

[B25] Maughan PJ Yourstone SM Jellen EN Udall JA SNP discovery via genomic reduction, barcoding and 454-pyrosequencing in amaranth Plant Gen 2009 2 260 270 10.3835/plantgenome2009.08.0022

[B26] Păcurar DI Păcurar ML Street N Bussell JD Pop TI Gutierrez L Bellini C A collection of INDEL markers for map-based cloning in seven *Arabidopsis* accessions J Exp Bot 2012 63 2491 2501 10.1093/jxb/err422 22282537 PMC3346218

[B27] Althoff DM Gitzendanner MA Segraves KA The utility of amplified fragment length polymorphisms in phylogenetics: a comparison of homology within and between genomes Syst Biol 2007 56 477 484 10.1080/10635150701427077 17562471

[B28] Sambrook J Fritcsh EF Maniatis T Molecular Cloning: A Laboratory Manual 1989 Cold Spring Harbor, N.Y: Cold Spring Harbor Lab

[B29] Todd JJ Vodkin LO Duplications that suppress and deletions that restore expression from a chalcone synthase multigene family Plant Cell 1996 8 687 699 12239396 10.1105/tpc.8.4.687 PMC161129

[B30] Holmgren NH Cronquist A, Holmgren AH, Holmgren NH, Reveal JL, Holmgren PK Penstemon Intermountain Flora: Vascular Plants of the Intermountain West. Volume 4 1984 Bronx, New York, USA: New York Botanical Garden 370 457

[B31] Welsh SL Atwood ND Goodrich S Higgins LC A Utah Flora 2008 4 Provo, Utah: Brigham Young University

[B32] RepeatMasker [http://www.repeatmasker.org]

[B33] Bao Z Eddy SR Automated de novo identification of repeat sequence families in sequenced genomes Genome Res 2002 12 1269 1276 10.1101/gr.88502 12176934 PMC186642

[B34] Price AL Jones NC Pevzner PA De novo identification of repeat families in large genomes Bioinformatics 2005 21 Suppl 1 I351 I358 10.1093/bioinformatics/bti1018 15961478

[B35] Maughan PJ Yourstone SM Byers RL Smith SM Udall JA Single-nucleotide polymorphism genotyping in mapping populations via genomic reduction and next-generation sequencing: proof-of-concept Plant Gen 2010 3 1 13

[B36] Rhee SY Beavis W Berardini TZ Chen GH Dixon D Doyle A Garcia-Hernandez M Huala E Lander G Montoya M Miller N Mueller LA Mundodi S Reiser L Tacklind J Weems DC Wu YH Xu I Yoo D Yoon J Zhang PF The Arabidopsis Information Resource (TAIR): a model organism database providing a centralized, curated gateway to *Arabidopsis* biology, research materials and community Nucleic Acids Res 2003 31 224 228 10.1093/nar/gkg076 12519987 PMC165523

[B37] Thiel T Michalek W Varshney RK Graner A Exploiting EST databases for the development and characterization of gene-derived SSR-markers in barley (*Hordeum vulgare* L.) Theor Appl Genet 2003 106 411 422 12589540 10.1007/s00122-002-1031-0

[B38] Stajich JE Block D Boulez K Brenner SE Chervitz SA Dagdigian C Fuellen G Gilbert JGR Korf I Lapp H Lehväslaiho H Matsalla C Mungall CJ Osborne BI Pocock MR Schattner P Senger M Stein LD Stupka E Wilkinson MD Birney E The Bioperl toolkit: Perl modules for the life sciences Genome Res 2002 12 1611 1618 10.1101/gr.361602 12368254 PMC187536

[B39] Rozen S Skaletsky HJ Krawetz S, Misener S Primer3 on the WWW for general users and for biologist programmers Bioinformatics Methods and Protocols: Methods in Molecular Biology 2000 Totowa, NJ: Humana Press 365 386 10.1385/1-59259-192-2:365 10547847

[B40] PAUP* Phylogenetic analysis using parsimony (*and other methods) [http://paup.csit.fsu.edu/]

[B41] Altschul SF Gish W Miller W Myers EW Lipman DJ Basic local alignment search tool J Mol Biol 1990 215 403 410 2231712 10.1016/S0022-2836(05)80360-2

[B42] GenBank [http://www.ncbi.nlm.nih.gov/genbank/]

[B43] Conesa A Götz S García-Gómez JM Terol J Talón M Robles M Blast2GO: a universal tool for annotation, visualization and analysis in functional genomics research Bioinformatics 2005 21 3674 3676 10.1093/bioinformatics/bti610 16081474

[B44] Kawabe A Miyashita NT Patterns of codon usage bias in three dicot and four monocot plant species Genes Genet Syst 2003 78 343 352 10.1266/ggs.78.343 14676425

[B45] Morgante M Hanafey M Powell W Microsatellites are preferentially associated with nonrepetitive DNA in plant genomes Nat Genet 2002 30 194 200 10.1038/ng822 11799393

[B46] Robinson JP Harris SA Gillet EM Amplified fragment length polymorphisms and microsatellites: a phylogenetic perspective EU-Compendium: Which DNA Marker for Which Purpose? 1999 Göttingen, Germany: Institut für Forstgenetik und Forstpflanzenzüchtung, Universität Göttingen 95 121

[B47] Ochieng JW Steane DA Ladiges PY Baverstock PR Henry RJ Shepherd M Microsatellites retain phylogenetic signals across genera in eucalypts (Myrtaceae) Genet Mol Biol 2007 30 1125 1134 10.1590/S1415-47572007000600016

[B48] Nadir E Margalit H Gallily T Ben-Sasson SA Microsatellite spreading in the human genome: evolutionary mechanisms and structural implications Proc Natl Acad Sci USA 1996 93 6470 6475 10.1073/pnas.93.13.6470 8692839 PMC39047

[B49] Viehmeyer G Reports dealing in large part with hybridization and selection Bul Amer Penstemon Soc 1965 24 95 100

[B50] Zamir D Tadmor Y Unequal segregation of nuclear genes in plants Bot Gaz 1986 147 355 358 10.1086/337602

[B51] Eshed Y Zamir D A genomic library of Lycopersicon pennellii in L. esculentum: A tool for fine mapping of genes Euphytica 1994 79 175 179 10.1007/BF00022516

[B52] Robbins MD Masud MAT Panthee DR Gardner RG Francis DM Stevens MR Marker assisted selection for coupling phase resistance to *Tomato spotted wilt virus* and *Phytophthora infestans* (late blight) in tomato HortScience 2010 45 1424 1428

[B53] Canady MA Meglic V Chetelat RT A library of *Solanum lycopersicoides* introgression lines in cultivated tomato Genome 2005 48 685 697 10.1139/g05-032 16094436

[B54] Canady MA Ji YF Chetelat RT Homeologous recombination in *Solanum lycopersicoides* introgression lines of cultivated tomato Genetics 2006 174 1775 1788 10.1534/genetics.106.065144 17057228 PMC1698654

[B55] Temnykh S DeClerck G Lukashova A Lipovich L Cartinhour S McCouch S Computational and experimental analysis of microsatellites in rice (*Oryza sativa* L.): frequency, length variation, transposon associations, and genetic marker potential Genome Res 2001 11 1441 1452 10.1101/gr.184001 11483586 PMC311097

[B56] Parida SK Kalia SK Kaul S Dalal V Hemaprabha G Selvi A Pandit A Singh A Gaikwad K Sharma TR Srivastava PS Singh NK Mohapatra T Informative genomic microsatellite markers for efficient genotyping applications in sugarcane Theor Appl Genet 2009 118 327 338 10.1007/s00122-008-0902-4 18946655

[B57] Zhang FK Zhao ZM The influence of neighboring-nucleotide composition on single nucleotide polymorphisms (SNPs) in the mouse genome and its comparison with human SNPs Genomics 2004 84 785 795 10.1016/j.ygeno.2004.06.015 15475257

[B58] Morton BR Bi IV McMullen MD Gaut BS Variation in mutation dynamics across the maize genome as a function of regional and flanking base composition Genetics 2006 172 569 577 16219784 10.1534/genetics.105.049916 PMC1456184

[B59] Schmuths H Meister A Horres R Bachmann K Genome size variation among accessions of *Arabidopsis thaliana* Ann Bot 2004 93 317 321 10.1093/aob/mch037 14724121 PMC4242198

[B60] Lynch M The Origins of Genome Architecture 2007 Sunderland, MA: Sinauer Associates, Inc

[B61] Lynch M Conery JS The origins of genome complexity Science 2003 302 1401 1404 10.1126/science.1089370 14631042

[B62] Kidwell MG Transposable elements and the evolution of genome size in eukaryotes Genetica 2002 115 49 63 10.1023/A:1016072014259 12188048

[B63] Raskina O Barber JC Nevo E Belyayev A Repetitive DNA and chromosomal rearrangements: speciation-related events in plant genomes Cytogenet Genome Res 2008 120 351 357 10.1159/000121084 18504364

[B64] Kolano B Gardunia BW Michalska M Bonifacio A Fairbanks D Maughan PJ Coleman CE Stevens MR Jellen EN Maluszynska J Chromosomal localization of two novel repetitive sequences isolated from the *Chenopodium quinoa* Willd. genome Genome 2011 54 710 717 10.1139/g11-035 21848446

[B65] Kubis S Schmidt T Heslop-Harrison JS Repetitive DNA elements as a major component of plant genomes Ann Bot 1998 82 Suppl A 45 55

[B66] Meyers B A summary of Bruce Meyers’ *Penstemon* hybridizations Bul Amer Penstemon Soc 1998 57 2 11

[B67] Friedt W Snowdon RJ Ordon F Ahlemeyer J Plant breeding: assessment of genetic diversity in crop plants and its exploitation in breeding Prog Bot 2007 68 151 178 10.1007/978-3-540-36832-8_7

[B68] Wolfe AD Elisens WJ Diploid hybrid speciation in *Penstemon* (Scrophulariaceae) revisited Amer J Bot 1993 80 1082 1094 10.2307/2445754 PMC20222 9560237

[B69] Wolfe AD Elisens WJ Nuclear ribosomal DNA restriction site variation in *Penstemon* section *Peltanthera* (Scrophulariaceae): an evaluation of diploid hybrid speciation and evidence for introgression Amer J Bot 1994 81 1627 1635 10.2307/2445341

[B70] Wolfe AD Elisens WJ Evidence of chloroplast capture and pollen-mediated gene flow in *Penstemon* sect. *Peltanthera* (Scrophulariaceae) Syst Bot 1995 20 395 412 10.2307/2419800

[B71] Datwyler SL Wolfe AD Phylogenetic relationships and morphological evolution in *Penstemon* subg. *Dasanthera* (Veronicaceae) Syst Bot 2004 29 165 176 10.1600/036364404772974077

[B72] Wolfe AD Xiang Q-Y Kephart SR Assessing hybridization in natural populations of *Penstemon* (Scrophulariaceae) using hypervariable intersimple sequence repeat (ISSR) bands Mol Ecol 1998 7 1107 1125 10.1046/j.1365-294x.1998.00425.x 9734070

[B73] Wolfe AD Xiang Q-Y Kephart SR Diploid hybrid speciation in *Penstemon* (Scrophulariaceae) Proc Natl Acad Sci USA 1998 95 5112 5115 10.1073/pnas.95.9.5112 9560237 PMC20222

[B74] Elshire RJ Glaubitz JC Sun Q Poland JA Kawamoto K Buckler ES Mitchell SE A robust, simple genotyping-by-sequencing (GBS) approach for high diversity species PLoS One 2011 6 e19379 10.1371/journal.pone.0019379 21573248 PMC3087801

